# The Role of Exhaled Breath Condensate in Chronic Inflammatory and Neoplastic Diseases of the Respiratory Tract

**DOI:** 10.3390/ijms25137395

**Published:** 2024-07-05

**Authors:** Karolina Kita, Marika Gawinowska, Marta Chełmińska, Marek Niedoszytko

**Affiliations:** Department of Allergology, Medical University of Gdansk, 80-210 Gdansk, Poland; karolina.kita@gumed.edu.pl (K.K.); marika.gawinowska@gumed.edu.pl (M.G.); allergy@gumed.edu.pl (M.C.)

**Keywords:** exhaled breath condensate (EBC), asthma, COPD, lung cancer, NSCLC

## Abstract

Asthma and chronic obstructive pulmonary disease (COPD) are among the most common chronic respiratory diseases. Chronic inflammation of the airways leads to an increased production of inflammatory markers by the effector cells of the respiratory tract and lung tissue. These biomarkers allow the assessment of physiological and pathological processes and responses to therapeutic interventions. Lung cancer, which is characterized by high mortality, is one of the most frequently diagnosed cancers worldwide. Current screening methods and tissue biopsies have limitations that highlight the need for rapid diagnosis, patient differentiation, and effective management and monitoring. One promising non-invasive diagnostic method for respiratory diseases is the assessment of exhaled breath condensate (EBC). EBC contains a mixture of volatile and non-volatile biomarkers such as cytokines, leukotrienes, oxidative stress markers, and molecular biomarkers, providing significant information about inflammatory and neoplastic states in the lungs. This article summarizes the research on the application and development of EBC assessment in diagnosing and monitoring respiratory diseases, focusing on asthma, COPD, and lung cancer. The process of collecting condensate, potential issues, and selected groups of markers for detailed disease assessment in the future are discussed. Further research may contribute to the development of more precise and personalized diagnostic and treatment methods.

## 1. Introduction

Chronic respiratory diseases are among the most prevalent non-communicable diseases worldwide, primarily due to harmful environmental, occupational, and behavioral factors. According to data available in 2017, approximately 545 million people worldwide were affected by chronic respiratory diseases, representing a 39.8% increase compared with 1990 [1]. Pneumonia, asthma, chronic obstructive pulmonary disease (COPD), lung cancer, and tuberculosis are the five most prevalent lung diseases worldwide [2].

As a result of chronic inflammation in the airways, there is an increased production of inflammatory markers by the effector cells of the airways and lung tissue. Experts from the National Institutes of Health define a biomarker as a characteristic that can be measured using objective methods, allowing the assessment of both physiological and pathological biological processes, as well as the body’s response to therapeutic processes [3]. In subsequent years, the Food and Drug Administration and the National Institutes of Health (FDA-NIH) Biomarker Working Group BEST (Biomarkers, Endpoints, and Tools) updated the definition, describing a biomarker as “a defined characteristic that is measured as an indicator of normal biological processes, pathogenic processes, or responses to exposure or intervention, including therapeutic interventions” [4].

Currently, the concept of biochemical phenotyping and the search for a connection between biochemical pathways and clinical symptoms have become the subject of broad discussion, not only for asthma but also for obstructive pulmonary disease (COPD) [5,6]. In recent years, proteomics and molecular research has focused on the identification of markers in the early stages of lung cancer development [7]. Lung cancer has dominated the statistics of incidence and death due to neoplastic diseases for many years. Therefore, there is a need to optimize and enhance screening methods using low-dose computed tomography. This is in order to accurately identify patients who require additional diagnostic assessment for lung nodules and establish a diagnosis through accessible, reproducible, and non-invasive means. The goal is to accurately identify patients requiring additional diagnostics for lung nodules and make the diagnosis using available, repeatable, and non-invasive methods.

This review summarizes the previous research on the utilization and advancement of exhaled breath condensate (EBC) assessment in the diagnosis of respiratory diseases. The focus has been on the most clinically relevant health issues in the population, including obstructive diseases (asthma and COPD) and neoplastic diseases (lung cancer).

## 2. Cell Stress Is Part of the Puzzle in the Pathogenesis of Chronic Lung Diseases

**Figure 1:**
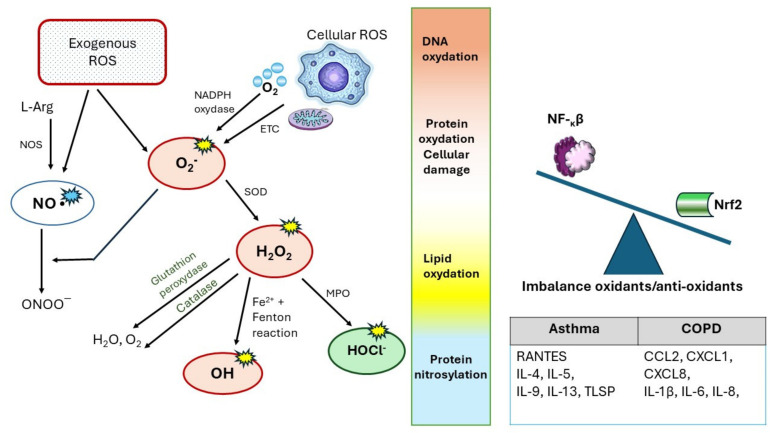
Oxidative-nitrosative stress pattern and examples of proinflammatory factors in asthma and COPD. Reagents based on oxygen are depicted in red, whereas those based on nitrogen are represented in blue. The green oval denotes the chlorine-based reagent, hypochlorous acid (HOCl). ETC = electron transport chain, Fe^2+^ = ferrous ion, HOCl^−^ = hypochlorous acid, H_2_O_2_ = hydrogen peroxide, L-Arg = L-arginine, MPO = myeloperoxidase, NO• = nitric oxide, NOS = nitric oxide synthase, ONOO^−^ = peroxynitritate anion, O_2_• = superoxide anion, OH• = hydroksyl radical, NADPH = nicotinamide adenine dinucleotide phosphate, NF-κβ = nuclear factor κβ (NF-κβ), Nrf2 = nuclear factor erythroid 2-related factor 2, ROS = reactive oxygen species, SOD = superoxide dismutase, RANTES = regulated upon Activation, Normal T Cell Expressed and Presumably Secreted, CXCL = C-X-C motif chemokine ligand, TLSP = thymic stromal lymphopoietin, IL = interleukin, NOS = nitric oxide synthase.

Reactive oxygen species (ROS) are generated in almost every intracellular organelle [8,9,10,11]. The first group of ROS, primarily derived from the mitochondria, is a byproduct of natural biological processes (Figure 1). Their production ultimately leads to the neutralization through repair mechanisms. Neutralization processes depend on enzymes such as superoxide dismutases, catalase, and peroxiredoxins, as well as antioxidant complexes, thioredoxin, and glutathione systems. The second group consists of ROS products generated through targeted production influenced by numerous enzymes, including the highly potent phagocytic nicotinamide adenine dinucleotide phosphate (NADPH) oxidase, NOX2, among others, as a component of the innate immune system [12,13,14]. Other ROS-producing enzymes include lipoxygenases and cyclooxygenases. The end-products of ROS are deliberately generated within the cell, leading to precise biological actions. The ROS system is thought to be functionally connected to intracellular organelles, potentially forming an integrated interorganellar network that regulates cellular homeostasis. Furthermore, it identifies stress states and activates appropriate responses including proliferation, inflammation, and apoptosis [15,16]. 

Physiologically, inflammation is a protective response to cellular and tissue damage that stimulates repair processes by eliminating harmful stimuli and damaged tissues. In situations where inflammation is uncontrolled, an excessive inflammatory response develops, causing cellular damage, tissue disruption, and consequently, a chronic inflammatory process [17]. 

**Figure 2:**
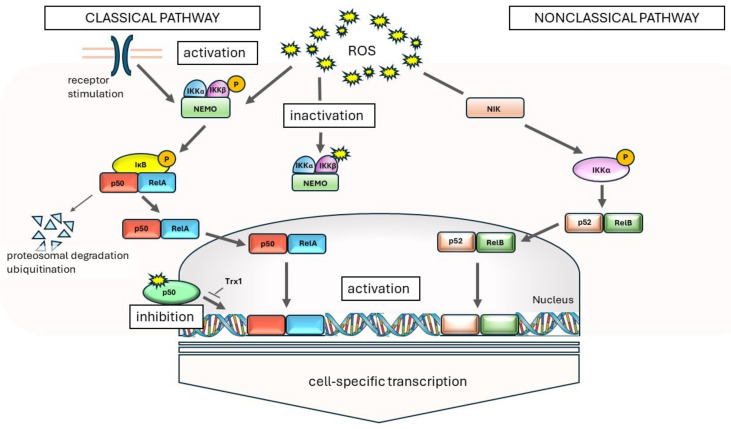
Scheme illustrating the influence of ROS on the activation of the NF-κB signaling pathway. The nuclear factor κβ (NF-κB) family comprises five subunits, which are NF-κB1 (p105/p50), NF-κB2 (p100/p52), RelA (p65), RelB, and cREL. The canonical NF-κB pathway is primarily activated by proinflammatory receptor stimulation, such as the tumor necrosis factor (TNF) family. These receptors activate the canonical signaling cascade by activating the kinases IκB (IKK) complex composed of IKKα and IKKβ (catalytic kinases) and the regulatory subunit IKKγ (NF-κB essential modulator, NEMO). As a result of ubiquitination and proteasome degradation, the heterodimer p50/RelA is released, undergoes nucleocytoplasmic transport, binds to κB sites, and activates the transcription of the gene. Noncanonical NF-κB activation is associated with the inducible kinase NF-κB (NIK). The stabilization of NIK leads to further noncanonical signaling events. Phosphorylation of p100 by IKKα, followed by ubiquitination and proteasomal degradation, allowing the release of p52 and its partner for DNA binding in the nucleus. ROS = reactive oxygen species, P = phosphate group, IκB = inhibitory protein kappa B, Trx = thioredoxin-1.

Under the influence of inflammation, leukocytes and respiratory epithelial cells release ROS and nitrogen species (RNS), leading to the disruption of intracellular redox processes and loss of the balance between oxidants and antioxidants, resulting in oxidative stress (OS). The oxidant–antioxidant system is further disrupted by exogenous oxidants present in substances, such as cigarette smoke (CS) and air pollution. The loss of homeostasis can lead to the inactivation of antiproteases, epithelial damage, neutrophil sequestration, and migration in the lungs, as well as the expression of inflammatory mediator genes. ROS influence nuclear factor κβ (NF-κβ) signaling pathways at various levels, and their interactions are complex and specific to the cell type [12,13,14]. NF-κB proteins are transcription factors that play a crucial role in inflammatory and immune responses. Moreover, these transcription factors regulate the expression of genes involved in cell growth, differentiation, development, and apoptosis. Typically, NF-κB activity is controlled by inhibitory kappa B (IκB) proteins. The activity of typical IκB is controlled by phosphorylation via upstream kinases IκB (IKK). There are several ways to activate NF-κB, and two main signaling pathways leading to NF-κB target gene activation have been described. These are referred to as the classical (or canonical) and alternative (or noncanonical) pathways. These two pathways can usually be distinguished based on which product participates in the NF-κB activation pathway: the canonical (p50/p105) or noncanonical (p52/p100) product. It is worth noting that p50 is often associated with RelA, while p52 is often associated with RelB [14]. The activation of the classical and nonclassical NF-κB pathways is illustrated in Figure 2. 

ROS can frequently operate in multiple locations within a given pathway, sometimes in a bilateral manner, which can complicate the clear influence of ROS on the NF-κB pathway. This may result from various upstream pathways and specific differences among cells [14]. 

In contrast, nuclear factor erythroid 2-related factor 2 (Nrf2) regulates antioxidant signaling pathways in cellular responses to OS. Upon the detection of oxidative stress, Nrf2 is phosphorylated (usually sequestered in the cytoplasm by Keap1), followed by the dissociation and translocation of Nrf2 to the cell nucleus. This interaction leads to the increased expression of antioxidant genes, inhibition of OS, and maintenance of redox homeostasis. In the presence of a predominance of OS and proinflammatory factors, including transforming growth factor β (TGF-β), Nrf2 is inhibited, and this antioxidant potential is suppressed [18]. 

## 3. Main Aspects of Exhaled Breath Condensate Methodology 

### 3.1. What Is Exhaled Breath Condensate?

Approximately 30 years ago, a new technique for sampling biological material from lungs emerged: exhaled breath condensate (EBC). According to the Task Force definition supported by the European Respiratory Society and American Thoracic Society (TF ERS/ATS), EBC is a diluted solution comprising various biomarkers with diverse chemical stability. EBC is collected into a condenser made of a chemically neutral material during tidal breathing over a specified time. The temperature range at which cooling takes place depends on the type of EBC collection device used and can range from 0 to below −20 degrees Celsius. It is recommended to use a nose clip and saliva trap [19]. Upon cooling, the exhaled air condenses, forming a liquid that serves as a sample from which numerous substances can be detected. The collected and condensed material contains volatile and non-volatile compounds and consists primarily of nitrogen, oxygen, carbon dioxide, argon, and water vapor. EBC is a relatively simple matrix, typically composed of over 99% water, formed through the condensation of exhaled humidified air. 

Volatile compounds dissolved in water were absorbed by condensing the water during sample collection. Breath metabolomics (breathomics) assumes that the profile of volatile organic compounds (VOCs) in exhaled air changes from the physiological to the pathological phase [20]. Each exhaled breath contains thousands of VOCs. VOCs are believed to originate from the environment (exogenous sources); the respiratory system itself (endogenous sources); and the oral, lung, and gut microbiomes [21,22]. Costello et al. highlighted that most known VOCs in exhaled air originate primarily from external sources, including local volatile substances in the air as well as byproducts of dietary and/or medication metabolism [23,24]. However, a significant focus of the current research is the fraction of endogenous and microbiological VOCs and their complex interactions within the metabolome [25]. 

Only a small portion of breath condensate consists of droplets of varying sizes containing non-volatile molecules, both insoluble and water-soluble. During tidal breathing, the released aerosol particle count ranges from 0.1 to 4 particles·cm^−3^ with an average diameter of 0.3 μm [26,27,28]. The trace concentrations of substances in the condensate pose a challenge for the development of standards for sample acquisition methodologies and biomarker identification. 

The process of the aerosolization of the lining fluid of the respiratory tract occurs through several mechanisms. Turbulent airflow through airways with sufficient energy to detach particles from the airway walls is considered the predominant mechanism [29]. During normal breathing, turbulence mainly occurs along the walls of the bronchi and trachea, as well as in the initial generations of bronchial branches, where cartilaginous rings modify the airflow. Turbulent flow also occurs in areas where there is a change in airflow direction, including the larynx and throat. In the respiratory system, the flow in the respiratory tract, which is a system of interconnected tubes, is neither exclusively laminar nor exclusively turbulent. Taking this fact into account, the flow in the respiratory tract can be indirectly defined by the Reynolds number, the values of which determine the limit of the formation of turbulent flow. This number is the ratio of inertial forces to viscous forces, and depends, among other things, on the velocity of the gas flow and is directly proportional to it. During physical exercise, the flow velocity in the respiratory tract increases, which generates turbulent flow. Physical exertion serves as a factor that increases the efficiency of aerosolization and droplet size, which through increased ventilation leads to the accumulation of energy required for particle detachment [27]. In contrast to the turbulence concept, another mechanism that occurs without the involvement of bulk flow has been proposed. According to Kharitonov et al., aerosolization of the lining fluid of the airways occurs when previously closed bronchioles and lung alveoli are opened [30,31]. 

### 3.2. Exhaled Breath Condensate Sampling

The feasibility of studying biomarker concentrations is significantly limited by the invasive methods of respiratory tract material sampling, which often cause discomfort to the patient. The primary advantages of collecting EBC samples include complete non-invasiveness, simplicity of execution, and the ability to perform multiple tests in virtually any environment. There is no need for medication administration or additional external fluid, typically added to the airways in procedures such as bronchoalveolar lavage (BAL). The EBC sampling procedure can be conducted in actively breathing patients of all ages and individuals on mechanical ventilation by connecting to the expiratory circuit of the ventilator [32]. To date, no adverse effects of the EBC sampling procedure have been documented in patients with lung diseases, both in the adult and pediatric populations, including severe lung conditions [33,34]. In addition, EBC collection did not trigger bronchial hyperreactivity. Only cases of participants exhibiting transient hyperventilation tendencies without adverse effects have been reported [19]. After 10 min of quiet tidal breathing in adults, 1–3 mL of EBC (air volume, V’ = 100 L/min) was collected, which provided a sample sufficient for analyzing numerous biomarkers.

The volume of condensate produced during exhalation varied among individuals. It was assumed that the size of the EBC sample depends on the minute volume under constant conditions in the condenser [35]. Low airflow rates during sampling are advantageous because, as the exhalation flow rate increases, the efficiency of the EBC collection procedure decreases [36] owing to increased dead space ventilation. The dead space ventilation is defined as the volume of air that is ventilated but does not participate in gas exchange. It includes the anatomical space, which consists of the nose, trachea, and bronchi, as well as the dead space of the alveolar sacs, which includes the respiratory bronchioles, alveolar ducts, acinar sacs, and alveoli. Increased ventilation may result in dilution of the EBC sample with a fraction originating from the dead space and a fraction from the inhaled surrounding air [35,37]. Coughing (spontaneous or induced) and crying during sampling may influence the composition of EBC [38,39].

Another factor affecting the quality of the collected condensate is the breathing path, that is, breathing through the nose and/or mouth. During nasal inhalation, the collected samples can potentially be contaminated through several mechanisms, including mixing of biomarkers from the nasal epithelium, nasal secretion drainage into the bronchi, and mixing of the nasal air fraction with the bronchial fraction. Existing reports indicate significant differences in the exhaled biomarker assessments of EBC between nasal and oral breathing [40,41]. Therefore, the use of a nose clip is recommended when breathing through the mouth. The quality of the collected sample also depends on saliva contamination, which can be minimized by using a mouthpiece with a saliva trap and recommending periodic swallowing during sampling. Variability in EBC quality is also attributed to external factors such as ambient temperature, relative humidity, and environmental pollutants. It is hypothesized that ambient air might influence the EBC composition, with inhaled mediators potentially reacting with molecules in the EBC or triggering inflammatory and/or immunological reactions within the respiratory tract [35,42,43]. Tobacco smoking remains a significant documented factor that modifies the content of the EBC condensate and affects the concentrations of specific biomarkers [19,44,45]. Attention should be paid to the increasing trend of waterpipe and electronic nicotine delivery systems’ consumption [46,47,48].

## 4. The Significance of Exhaled Breath Condensate Analysis and Its Clinical Implications in Selected Respiratory Diseases

### 4.1. Asthma

Asthma is a heterogeneous disease characterized by concurrent chronic airway inflammation. Respiratory symptoms are accompanied by wheezing, shortness of breath, chest tightness, coughing, and variable airflow limitation [49]. It is a chronic respiratory disease with an estimated prevalence of 1–29% of the population [50,51]. The pathogenesis of asthma involves complex interactions between environmental, epigenetic, and genetic factors that shape diverse disease processes. As a result, a complex spectrum of clinical features shapes asthma phenotypes (Table 1). Therefore, there is a need to identify a broad array of biochemical and molecular pathways underlying the diversity of asthma [52,53]. 

Specific cytokine profiles are associated with specific inflammatory states in the airways of patients with asthma, and metabolic profiling can facilitate the definition of the disease phenotype. The foundation of the rapid development of breathomics lies in numerous studies analyzing factors that trigger airway inflammation and identifying markers of nitrosative and OS in EBC samples collected from patients with asthma [35,36,59,60].

Currently, a multicenter, prospective study titled “Precision Medicine Intervention in Severe Asthma (PRISM) study: molecular phenotyping of patients with severe asthma and response to biologics” (NCT05164939) encompasses a cohort of patients with severe asthma [61]. The recorded data include both clinical information and comprehensive multiomic data derived from various biological materials, including EBC from asthma patients with T2-high and T2-low phenotypes. The aim of this study was to explore new molecular phenotypes and responses to treatment, including biological therapies.

#### 4.1.1. Acidity (pH) Measurement

The role of the EBC pH as a candidate prognostic factor for asthma remains unclear. Some studies have shown a significant decrease in pH in patients with stable asthma [62] compared with healthy individuals, with further reductions observed in patients with asthma exacerbations [63,64,65,66,67]. The acidification of the respiratory tract in EBC measurements correlated with eosinophilia in induced sputum and the intensity of oxidative and nitrosative stress [66] and normalized after treatment with inhaled corticosteroids (ICS) [63,67]. Based on these findings, it could be expected that low pH values would be observed in the group of patients with severe asthma, which was not confirmed in studies with large cohorts [68,69]. Furthermore, in the Severe Asthma Research Program study (n = 572), the EBC pH results were not lower in either the severe asthma group (8.02; interquartile range [IQR], 7.61–8.41) or the mild asthma group (7.90; IQR, 7.52–8.20) compared to the healthy control group (7.9; IQR 7.40–8.20). On the other hand, a group of participants with stable asthma and decreased pH < 6.5 was identified, among whom, after multiple linear regression analysis, a phenotype of asthma patients with concomitant obesity, predominant neutrophilic inflammation, and significant obstruction on spirometry was identified [70].

Hypothetically, the EBC pH values may be modulated by various factors, including comorbidities, anti-asthmatic treatment, respiratory tract infections, and tobacco smoking [19,71]. Comorbidities in asthmatic patients, such as rhinosinusitis and gastroesophageal reflux, often worsen the disease course [49]. A reduced EBC pH has been observed in children with allergic rhinitis [67]. Interestingly, concurrent gastroesophageal reflux did not significantly affect the EBC pH [72,73] and treatment with a proton pump inhibitor did not significantly alter the EBC pH [72]. 

Active smoking negatively affects the asthma course. Smoking has been reported to decrease the EBC pH [65]; however, despite including healthy smoking controls in studies, there is often a lack of information about smoking in the asthmatic group [74]. 

#### 4.1.2. Dysregulation of the Oxidant-Antioxidant Axis in Asthma

In vivo, a wide range of biochemical processes involve redox reactions, and the onset of OS and nitrosative stress (NS) may occur via endogenous and/or exogenous pathways. Both free radicals and other highly reactive oxidants that interact with microenvironment components give rise to a pool of highly active molecules, such as ROS and RNS, which mutually react. 

Activated inflammatory cells (eosinophils, neutrophils, and mast cells) within the bronchial walls are sources of inflammatory mediators and reinforce proinflammatory actions, leading to the increased production of ROS, including hydrogen peroxide (H_2_O_2_) [75,76]. The intricate inflammatory infiltrate in asthma does not distinctly identify the primary cellular source of H_2_O_2_ in EBC [74,77,78]. 

Hypothetically, as an inflammation biomarker, H_2_O_2_ may reflect the intensity of inflammation within the bronchial walls; however, the complexity of inflammatory infiltration in asthma [79] does not definitively indicate the primary cellular source of hydrogen peroxide in EBC [74,77,78]. Numerous studies have demonstrated increased EBC H_2_O_2_ levels in both adult and pediatric populations [80,81,82,83], including the cough variant asthma (CVA) phenotype [77]. H_2_O_2_ levels in EBC correlated with eosinophil counts in induced sputum, bronchial hyperreactivity in bronchial challenge tests [82,84], decreased forced expiratory volume in 1 s (FEV1) [75], and asthma symptom severity compared with the stable disease phase. Interestingly, one study demonstrated that OS within the respiratory tract did not significantly affect asthma symptom control, as assessed using the Asthma Control Test (ACT) [85]. The effects of the disease-modifying medications were also investigated. Treatment with glucocorticosteroids was found to significantly reduce H_2_O_2_ levels in EBC [86,87], whereas antileukotriene drugs did not exhibit such an effect [80]. A meta-analysis with a large sample size (n = 728) confirmed these findings [88]. 

Free radicals generated from OS induce non-enzymatic arachidonic acid peroxidation within cell membrane phospholipids [89,90], leading to the in vivo production of isoprostanes (IsoPs) [91]. Compared to other OS markers, IsoPs in EBC could potentially serve as reliable biomarkers; in vivo, they exhibit chemical stability and lipid peroxidation specificity [92,93]. The most extensively studied representative of this group is 8-isoprostane (8-isoP), which belongs to the F2 isoprostane class. Higher values of 8-isoP have been documented in patients with asthma in an invasive BAL study [94]. Numerous studies using EBC have revealed significantly higher concentrations of EBC 8-isoP in asthma groups than in healthy control groups [95,96,97,98], with levels increasing with asthma severity [96,99]. In one study, it was suggested that together with FENO, it may reflect inflammation of the small airways and, along with pulmonary spirometric evaluation, could potentially be useful in monitoring the course of asthma [100]. In a study by Baraldi et al., involving a pediatric asthma population, higher 8-isoP values were observed in children with asthma, including during exacerbations, with a decrease in this biomarker level after inflammatory treatment (oral prednisone). In a significant finding, it was observed that despite receiving oral glucocorticosteroid therapy, the levels of 8-isoP in EBC remained elevated in pediatric patients with asthma as compared to the control group. This suggests that glucocorticosteroids may not be completely successful in mitigating OS in children with asthma exacerbations [101]. 

Despite promising research results regarding EBC 8-isoP as a candidate for monitoring the course and treatment of asthma, there have been studies that did not show a correlation with this biomarker in asthma patient groups [102,103]. These findings may have limitations, likely owing to the limited power of the study sample. However, methods based on immunoassays, which have been widely used in numerous studies, should be validated using precise analytical techniques, such as liquid chromatography–mass spectrometry (LC-MS), to ensure quantitative compound analysis in EBC [19,104,105]. The findings of a meta-analysis that incorporated 52 studies on EBC 8-isoP suggest that the collection stage of EBC is a crucial factor affecting the measured concentrations of 8-isoP. It remains uncertain whether this difference originates from the exhalation collection device itself or from the non-uniform collection conditions of the condensate [105]. 

Another eicosanoid studied in asthmatic patients was prostaglandin E2 (PGE2) in EBC. In both pediatric and non-smoking adult groups with stable asthma, no significant increase in EBC levels has been observed [106,107]. Interestingly, further analysis revealed that PGE2 levels were significantly higher in smoking asthma patients than in both smoking and non-smoking control groups [107]. 

Endogenously produced nitric oxide (NO) coming from both endogenous respiratory and inflammatory cells plays a physiologically important role in the respiratory system. NO synthesis is catalyzed by three synthase NO (NOS) isoforms and is dependent on oxygen and nicotinamide adenine dinucleotide phosphate (NADPH). While NO generated from the constitutive NOS fraction (cNOS) plays a crucial role in maintaining hemostasis, such as the vasodilation of lung vessels and smooth muscle relaxation of the airways, in the pathogenesis of NS and airway inflammation, inducible NO derived from NOS2 plays a fundamental role [108]. The NO generated by NOS2 plays a key role in airway inflammation. Factors that stimulate NOS2 activity include tumor necrosis factor-alpha (TNF-α), interferon-gamma (IFN-γ), interleukin (IL)-1 beta (IL-1β), and lipopolysaccharide (LPS). During the development of tissue inflammation, superoxide anions may be concurrently produced. NO rapidly reacts with superoxide anions to form peroxynitrite (ONOO^−^), which is an RNS [109]. RNS can also be generated through the nitrite oxidation pathway, depending on the presence of H_2_O_2_ and peroxidases [110], contributing to airway inflammation [111]. Exhaled RNS in aerosol droplets during respiration can be detected in EBC. 

Studies have demonstrated an increase in the end products of NO metabolism (NOx) in EBC in the asthma patient group compared to the healthy control group [87,112]. Elevated concentrations correlate with worsening lung function, as measured by FEV1 [112] and EBC H_2_O_2_, a biochemical OS indicator [87,113]. Additionally, EBC NOx levels were significantly lower in adult asthma patients treated with ICS [87]. Notably, NOx levels exhibited considerable variability. In a study by Rihak et al., not only was variability observed in NOx levels in the healthy participant group, but the values in healthy participants were also higher than those in the group of patients with chronic lung conditions, likely due to sample contamination in EBC collection [112]. 

The role of the EBC NOx level as a reliable inflammatory marker for monitoring patients with asthma remains unclear. Further research, following the analysis of a large group in the Epidemiological Study on the Genetics and Environment of Asthma (EGEA) study (n = 523), did not show a relationship between EBC nitrite (NO_2_^−^) and nitrate (NO_3_^−^) levels and asthma [114]. In a large cohort study (n = 8583) evaluating subclinical inflammation in adult patients with rarely occurring spontaneous asthma remission (aged 45 and 50 years), no evidence of a significant correlation between EBC NOx and asthma relapse or worsened lung function was found [115].

The formation of peroxynitrite from the reaction of NO with superoxide anions (O_2_^−^) in the airways is a well-known phenomenon. This highly reactive oxidant can react with tyrosine residues in proteins to produce a stable product known as nitrotyrosine (NT) [116]. In one study, NT concentrations were detectable in EBC in both healthy individuals (6.3 ± 0.8 ng/mL) and individuals with mild asthma, with significantly higher levels observed in asthma (15.3 ± 2.0 ng/mL, *p* < 0.01); however, the severity of asthma did not correlate with EBC NT concentration [117]. Different results have been reported in other studies: no significant association of this biomarker in EBC with asthma was found in either the pediatric [118] or adult asthma patient groups [119]. 

#### 4.1.3. Leukotrienes

Cysteinyl leukotrienes (Cys-LTs) are potent bronchoconstrictors and proinflammatory mediators. They are produced as a result of arachidonic acid oxidation catalyzed by five types of lipoxygenases in effector cells at the site of inflammation: mast cells, eosinophils, basophils, and macrophages [120,121,122,123]. In numerous studies, eicosanoid levels have been assessed in serum and urine, with the results possibly reflecting systemic inflammation rather than pulmonary inflammation [124,125,126,127,128]. In a study by Lex et al., a significant correlation between Cys-LTs in EBC and basement membrane thickness was reported, indicating the role of Cys-LTs in airway remodeling [129]. 

Elevated levels of Cys-LTs have been detected in BAL fluid and induced sputum [122,123]. The level of leukotrienes in EBC is elevated in patients with asthma, especially in cases of unstable asthma [106,117,128]. In a study by Segovia et al., a higher level of Cys-LTs (LTC4, LTD4, and LTE4) in EBC was observed in both episodic and chronic asthma, with concentrations being higher in the latter group [130]. Furthermore, an increase in EBC Cys-LT levels among patients with exercise-induced bronchoconstriction (EIB) was observed in the EBC of EIB patients [131,132], and Cys-LT levels before exercise were higher in EBC patients with EIB (median concentration 42.2 pg/mL) than in those without EIB (11.7 pg/mL) [131]. These findings may reflect the involvement of Cys-LTs in the pathogenesis of EIB. 

Considering that airway inflammation in asthma affects both the large and small airways, potential differences in EBC biomarker concentrations may exist depending on airway diameter. In the study by Tischler et al., after analyzing fractionated EBC (using the Eco Screen2 condenser), no differences in LTB4 were found between healthy volunteers and patients with asthma in terms of large airways. However, in children with asthma and concurrent bronchial obstruction in spirometry, increased LTB4 levels were observed in small airways or lung alveoli compared to children without spirometric abnormalities (2.0 pg/mL; 95% IQR, 2.0–9.21 pg/mL; vs. 18.32 pg/mL, 95% IQR, 3.7–23.02 pg/mL, *p* = 0.04). Moreover, these values remained higher in the latter group than in the healthy control group. Thus, EBC LTB4 can be utilized as a non-invasive marker for diseases of the small airways [133]. Notably, LTB4 is not a specific marker for asthma, and a significant increase in EBC LTB4 has been documented in other chronic lung diseases [134,135,136]. In the study conducted by Kazani et al. on patients with asthma, higher concentrations of not only LTB4, but also Lipoxin A4 (LXA4) were found in EBC. The concentration of LXA4 demonstrated a strong correlation with the degree of obstruction in the airways, as measured by spirometry, and as the severity of asthma increased, the ratio of LXA4 to LTB4 decreased. The level of EBC LXA4 proved useful in the diagnosis of asthma, with two cutoff values established: an LXA4 cut-off value of 7 pg/mL in EBC and 11 pg/mL in EBC providing 90% and 100% sensitivity and 92% and 100% specificity, respectively [137]. 

Treatment with a single high dose of inhaled corticosteroids (fluticasone propionate) and oral corticosteroids (prednisone) significantly decreased Cys-LT concentrations in both the early phase of asthma exacerbation treatment and the late stage of therapy in patients with asthma exacerbations [132]. 

LTB4 is not a specific marker for asthma, and a significant increase in EBC LTB4 has been documented in other chronic lung diseases [134,135,136].

Among other factors influencing higher leukotriene values in the condensate are the coexistence of atopy [132,133] and exposure to passive smoking [130]. 

#### 4.1.4. Cytokines

In asthma, regulation of the inflammatory process involves IL-2, which stimulates T-lymphocyte proliferation [138]. It has been demonstrated that higher concentrations of IL-2 in the condensate are present in patients with asthma and are correlated with asthma severity. In non-allergic asthma patients, IL-2 levels showed an inverse correlation with ACT results and FEV1 values in spirometry [139]. 

In the classical asthma model, a key role is played by the Th2 helper T-cell subpopulation, which produces a characteristic cytokine profile: IL-4, IL-5, IL-13 [140,141]. Numerous studies have shown an increase in IL-4 levels in EBC [142,143,144,145,146]. Research conducted by Robroeks et al. determined that elevated levels of IL-4 in EBC were indicative of asthma in children, with IL-4 being the sole significant predictor of asthma diagnosis. Additionally, the study found that IL-4 EBC levels were linked to asthma control but did not correlate with disease severity [142]. Subsequent studies have revealed a negative correlation between the ICS dose and the IL-4 concentration in condensate [143]. Elevated IL-5 levels in EBC have also been recorded in patients with asthma [147], particularly in those with concomitant atopy [148]. Robroeks et al. reported that during a one-year observation in the FLAME study, IL-5 and EBC pH were prognostic factors for exacerbations in 40 asthmatic children [149]. These results contrast with another prospective study in which the presence of IL-5 in EBC varied widely, and the predictive power of IL-5 in EBC in predicting asthma exacerbations was low, even when combined with typical clinical symptoms [150]. Notably, EBC samples from children do not always contain detectable cytokines [151]. Similar contradictory results have been noted for IL-6 levels in EBCs. Higher values were observed in adult participants with asthma [145,152] and in the pediatric patient group [153]. Considering that external factors also influence the inflammatory state within the airways, a study by Duman et al. did not show a statistically significant difference in IL-6 EBC levels between non-smoking and tobacco-smoking patients with newly diagnosed asthma. Furthermore, treatment with ICS or ICS + long-acting beta-agonists (LABA) for three months also had no effect on IL-6 EBC [154].

Physical effort may also modulate the course of airway inflammation. In a prospective study involving asthma patients (n = 21) who underwent aerobic training for three months, there was a decrease in the levels of proinflammatory interleukins in EBC IL-1β (*p* = 0.0008), IL-4 (*p* = 0.0481), IL-5 (*p* < 0.0001), IL-6 (*p* = 0.0032), IL-13 (*p* = 0.0013), TNF-α, markers of remodeling (profibrotic), vascular endothelial growth factor (VEGF), and thymic stromal lymphopoietin (TSLP). The EBC results were consistent with the blood serum biomarker levels. Moreover, a reduction in eosinophil and macrophage numbers in induced sputum, as well as eosinophils in peripheral blood, indicates the modulation of inflammation in the airways at the tissue level [155].

Studies have also evaluated the EBC levels of IL-8 [156], IL-17 [157], and IL-13 [158]. Studies on IL-6 and IL-26 levels in EBC may be helpful for phenotyping asthma with concomitant obesity [159,160].

#### 4.1.5. Chemokines

CC chemokines, such as RANTES, facilitate the migration and activation of proinflammatory cells, including Th12 lymphocytes and eosinophils [161], and stimulate the release of histamine and Cys-LT from mast cells, as well as eosinophilic cationic protein (ECP), ultimately leading to bronchial constriction. CC chemokines have been identified in BAL and induced sputum [162,163]. Elevated levels of RANTES in EBC have been observed in individuals with asthma [164], particularly in steroid-naive patients with asthma [157,164] and those with unstable asthma [164]. RANTES EBC studies have shown an association between reduced FEV1 and increased airway resistance [157]. Moreover, a correlation between RANTES EBC levels in asthma patients with EIB has been confirmed [165], suggesting the involvement of RANTES in promoting the inflammatory process in the airways in this specific patient subgroup. Further investigations by Zietkowski et al. indicated that treatment with the biological agent omalizumab reduced RANTES levels in EBC patients with severe allergic asthma, contributing to the suppression of airway inflammation [166]. 

#### 4.1.6. Proteins

During the Th2 inflammatory response, released IL-4 and IL-13 induce the expression of periostin, a protein present in the extracellular matrix [167,168]. Periostin regulates collagen accumulation and fibrosis during the remodeling process [169]. In the airways, this mechanism translates to thickening of the basement membrane, fibrosis, and tissue eosinophilia in the nasal and sinus mucosa [170] and bronchi [171,172]. Studies involving adults with asthma have reported higher levels of periostin in EBC among asthma patients [173,174,175], particularly significantly elevated levels in those with Th2-type inflammation compared to those with non-Th2 asthma, with a direct correlation between periostin EBC levels and disease severity [174]. In another study, increased periostin levels in EBC were detected in patients with asthma with concurrent chronic inflammation of the nasal and sinus mucosa, including respiratory diseases exacerbated by non-steroidal anti-inflammatory drugs (NERDs) [55], especially in individuals with positive bacterial cultures in nasopharyngeal swabs [173]. 

In a pediatric group of patients with mild asthma, a low level of periostin in the EBC was found, with no differences between asthmatic individuals and the control group [176]. Studies have shown that the concentration of this protein increases during children’s skeletal growth, which may coincide with local production in the airways [177] and act as a confounding factor in the measurement of this biomarker in EBC. Therefore, periostin appears to be an unlikely biomarker for Th2-type inflammation in children with asthma. 

#### 4.1.7. MicroRNAs

MicroRNAs, also known as miRNAs, are small non-coding RNA (ncRNA) molecules that play a critical role in the regulation of gene expression. Specifically, they exert their effects by modulating various signal transduction pathways such as cell proliferation, differentiation, and apoptosis. MiRNAs are predicted to regulate approximately 60% of all human protein-coding genes [178]. In the pathomechanism of asthma, miRNAs interact with the smooth muscles, epithelium, and immune system in the airways, with the ultimate effects of these processes being remodeling, smooth muscle contraction, promotion of the Th2 response, and suppression of Th1 cytokine secretion [179]. The transport of miRNAs from host cells to target/effector cells occurs through extracellular vesicles (EVs), which can be effectively detected in the blood, urine, EBC, and BAL fluid [180]. To date, few studies have evaluated miRNAs in patients with EBC and asthma. 

The effective isolation of miRNAs from EBC has been previously reported. In a study conducted by Pinkerton et al., it was observed that the expression of miR-1248, miR-1291, Let-7, miR-328, and miR-21 was decreased in the EBC of asthma patients compared to the healthy control group. 

The studied miRNA, by interacting with target genes, participates in the regulation of IL-13 and/or IL-5 receptor functions, and the demonstrated decreased levels of miRNA in EBC may be associated with the dysregulation of the Th2 cytokine pathway in patients with asthma [181]. 

In one study, decreased expression of miR-570-3p in EBC was observed in patients with asthma, and this result was inversely correlated with lung function [182]. In the study by Roff et al., the authors suggested that miR-570-3p could be a potential biomarker for regulating the inflammatory state in patients with asthma due to diverse interactions with the expression of various cytokines, chemokines (CC chemokine ligand (CCL) 4, CCL5, TNFα, IL-6, CCL2, and IL-8), and the RNA-binding protein HuR, which is involved in post-transcriptional regulation [182]. 

In studies involving children with asthma, miRNA expression in EBC was shown to correlate with salbutamol-responsive asthma phenotypes [183]. Another study focusing on a pediatric population highlighted miRNA-423, whose association with obesity has been demonstrated [184]. 

MiR-328-3p is strongly linked to various inflammatory factors such as tumor TNF-α, IL-6, and IL-1β [185]. Additionally, by modulating TGF-β1, it regulates the proliferation, migration, and inflammation of smooth muscles in the airways, contributing to remodeling in asthma [186]. The seasonal variability of miR-328-3p has also been demonstrated to affect the seasonal symptoms observed in patients with asthma [187]. 

#### 4.1.8. The Respiratory Microbiota

Microbial diversity plays a significant role in the risk of developing asthma and the increasing trend of its prevalence, as a lack of microbiome enrichment favors the perpetuation of aberrant inflammatory pathways and allergic reactions [188]. Conversely, certain pathogens of viral origin have been shown to promote asthma exacerbation [189]. Previously, the lower airways were thought to be sterile [190]; however, the bronchial microbiome has not been characterized. In patients with asthma, qualitative and quantitative dysregulation of the bronchial microbiome is observed. An et al., leveraging an artificial intelligence (AI) model, demonstrated the dominant presence of Bacteroides and Firmicutes, where no significant associations were found with the levels of eosinophils and neutrophils in the sputum, asthma severity and duration, obesity, smoking, or ICS [191]. In a study evaluating the fungal microbiome in EBC, fungal colonization was identified in 70% of patients with asthma but was not detected in the healthy control group. Predominantly, Cladosporium (94% of asthma patients), Alternaria (21%), and Penicillium (24%) were identified. The microbiological findings were consistent with those of the induced sputum. Moreover, the presence of fungi was higher in patients with asthma without atopy, severe uncontrolled asthma, and concurrent obesity [192].

In the analysis of children with asthma, it was revealed that the respiratory microbiome exhibited greater species richness in EBC (Shannon diversity index, mean 3.029 vs. 2.642, *p* = 0.026) than in healthy children. At the phylum level, Firmicutes dominated, followed by Proteobacteria and Actinobacteria, whereas the classes Gammaproteobacteria and Bacillus in EBC were less abundant in the asthma group [193]. 

The findings from the current study underscore the necessity for further investigation into the relationship between dysbiosis and the pathogenesis and progression of asthma.

### 4.2. Chronic Obstructive Pulmonary Disease (COPD)

COPD is a common chronic respiratory condition characterized by airflow limitation, which is often progressive, and a lack of reversibility of the obstruction in spirometry [194,195].

COPD is the third most common cause of mortality globally, as reported by the World Health Organization (WHO) [196], and is also a major contributor to disabilities [196,197].

**Figure 3:**
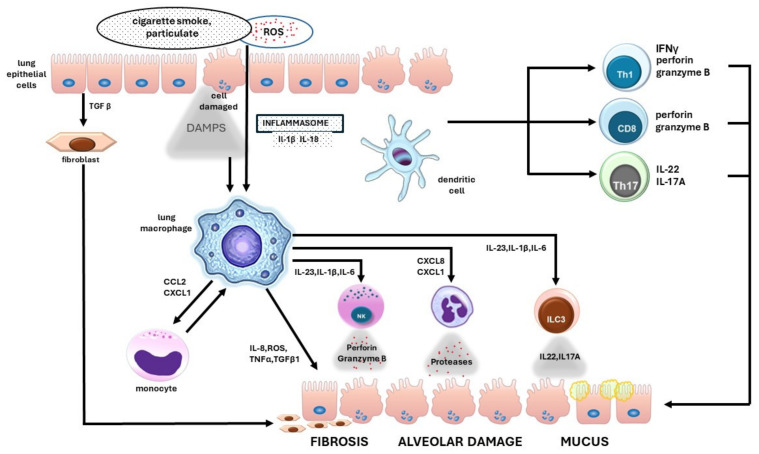
Activation of innate and adaptive immunity in COPD. ROS = reactive oxygen species; IL = interleukin, TGFβ = transforming growth factor β, DAMPS = damage-associated molecular patterns; IFNγ = Interferon gamma; CCL2 = CC chemokine ligand; CXCL = C-X-C motif chemokine ligand, TNFα = tumor necrosis factor-alpha.

The activation of pattern recognition receptors (PRRs) upon the recognition of particles (damage-associated molecular patterns—DAMPs) in inhaled air leads to the release of proinflammatory cytokines such as IL-1α, IL-1β, IL-33, and IL-18. Under the influence of these interleukins, neutrophils, macrophages, and Th1 and Th17 helper T cells are activated, resulting in Type 1 inflammation [198]. Macrophages play a crucial role in the development of respiratory inflammation (Figure 3). TNF-α secreted by macrophages promotes the expression of adhesion molecules in the endothelium, which facilitates and promotes inflammatory cell migration. Additionally, alveolar macrophages release ROS, proteases, cathepsins, and TGFβ, which leads to damage to the anatomical structure of the alveoli and causes fibrosis [199].

Under the influence of ROS, there is a concentration of 3,4,5-trifosphate of phosphatidylinositol at the site of injury, which stimulates neutrophil migration [200]. Additionally, neutrophil migration is enhanced by (1) macrophages through C-X-C motif chemokine ligand 1 (CXCL1), CXCL8, and LTB4 ligands; (2) Th cells; and (3) innate lymphoid cell 3 (ILC3) due to the release of IL-22 and IL-17A [201,202]. In the respiratory tract, neutrophils release elastase, neutrophil proteinase, and matrix metallopeptidase (MMP), which are associated with alveolar damage and the stimulation of mucus secretion [199].

Acquired immunity occurs through the recognition of damaged antigens by dendritic cells and the subsequent presentation of these fragments to T cells. In COPD, there is an increase in the Th17/Treg ratio in blood and sputum [203,204]. Th17 cells produce the proinflammatory cytokine IL-17 and can promote the activation of fibroblasts, epithelial cells, and smooth muscle cells in the trachea. Additionally, CD8-positive cytotoxic T cells and Th1 CD4-positive T cells release perforins and granzyme B, which enhance alveolar destruction [205,206].

The earliest described molecular mechanism in COPD pathogenesis was an imbalance in the proteolysis (elastase)–antiproteolysis (α1-antitrypsin inhibitor) axis. Consequently, elastase produced by neutrophils leads to severe panlobular emphysema in the presence of functional α1-antitrypsin deficiency (A1AD) [207]. A similar concept of disturbances in proteolytic–antiproteolytic systems has been associated with the lung tissue destruction and emphysema development induced by CS. Based on this assumption, inflammation stimulates both the secretion and hyperactivity of proteases, whereas OS suppresses the antiproteolytic barrier [208]. The complex interplay between exogenous factors (CS, environmental pollution, prior infections, occupational exposures) and endogenous factors (airway hyperreactivity, genetic predisposition, antioxidant defense potential, age, and sex) contributes to the heterogeneity of the pathomechanism underlying COPD development [209].

An imbalance between the generation of ROS/RNS and the compensatory capacity of antioxidant systems leads to OS/NS, potentially resulting in cellular component damage [210,211] and affecting proteins [212,213], lipids [214], and DNA [215]. These processes alter compound structures, their overall function, and activity changes [216,217,218], ultimately leading to disruptions in membrane function and abnormal proliferation and apoptosis [219,220,221]. ROS/RNS are primarily produced in lung tissue, originating from neutrophils, alveolar macrophages, and eosinophils, and their production also occurs in bronchial cells, alveolar epithelium, and the endothelium [222,223]. 

Disruption of the lung architecture in COPD may affect EBC sampling and sample quality. Research findings indicate that COPD patients exhale fewer particles than healthy controls [224]. Impaired aerosolization and exhaled particle formation may be associated with lung hyperinflation and expiratory disturbances in COPD patients. Furthermore, the destruction of terminal bronchioles and their reduced numbers in COPD can lead to disruptions in the opening and closing of distal airway segments.

#### 4.2.1. Acidity (pH) Measurement

It was hypothesized that measuring the pH of the EBC would allow the assessment of endogenous airway acidification, which could reflect inflammation and disease severity in COPD. A significant decrease in EBC pH was observed in patients with COPD [66,85,225], with lower pH values in GOLD stages III–IV compared to stage I [226] during acute exacerbations of COPD (AECOPD) [227], although the correlation with FEV1 was contradictory [66,85]. In comparison with asthma patients, the EBC pH was lower in COPD patients, and treatment with ICS, unlike asthma, did not show a significant correlation with the EBC pH, likely due to predominant neutrophilic inflammation [66]. Furthermore, tobacco smoking influences pH values, with this parameter being significantly reduced in chronic smokers (at least 10 pack years) compared to the control group [225].

Chronic colonization by pathogenic bacteria should also be considered when interpreting EBC pH results. Studies have demonstrated that the presence of pathogenic bacteria in pleural fluid [228], as well as the colonization of *P. aeruginosa* in bronchiectasis [66,229], significantly lowers pH, suggesting a similar effect within the airways of COPD patients with colonization by *P. aeruginosa*.

#### 4.2.2. Markers of Oxidative and Nitrosative Stress

Neutrophils play a major role in COPD pathogenesis. It is well known that an increased number of neutrophils correlates with elevated ROS levels [230]. Thus, the high proportion of these cells in sputum and the strong association between neutrophils and H_2_O_2_ EBC levels in EBC suggest that neutrophilic cells are the primary source of H_2_O_2_ in patients with COPD [37].

Several studies have assessed the potential of H_2_O_2_ as a biomarker of inflammation in COPD; however, its significance remains unclear. Initially, elevated concentrations of H_2_O_2_ in exhaled air were observed in COPD patients compared to healthy individuals [37,231,232], and significant correlations were reported not only with disease severity [37,231] but also with FEV1 values, neutrophil counts in induced sputum, and dyspnea severity on the Medical Research Council (MRC) scale [37]. Additionally, the level of H_2_O_2_ in EBC correlates with the severity of symptoms and risk of COPD exacerbations, assessed using the MRC scale [37] and CAT [85].

Chronic COPD treatment and COPD exacerbation showed variable correlations with H_2_O_2_ levels in EBC patients. In one study, it was found that ICS treatment did not affect H_2_O_2_ concentrations in EBC [37]. Conversely, in other studies, inhaled ICS [233,234] and antibiotic therapy in infectious exacerbations [234] reduced H_2_O_2_ values in EBC, while these values remained elevated in patients with stable COPD compared to healthy individuals. This could be an effect, on the one hand, of reducing the number of inflammatory cells and, on the other hand, of simultaneously and constantly activating proinflammatory processes. In patients with COPD, neutrophils are the main source of H_2_O_2_ in the airway. Neutrophilic inflammation and concomitant impaired antioxidant efficiency lead to the inefficacy of ICS treatment in patients with stable COPD [235,236]. 

The results of a study by Montuschi et al. indicated that 8-isoP could induce bronchial constriction in vitro [237]; hence, this parameter has been extensively studied in obstructive diseases. Increased values of this biomarker in exhaled breath have been reported in patients with COPD [238], as well as a rise in levels during COPD exacerbations [234,239,240]. It is important to emphasize that despite the higher values of 8-isoP in air condensate, no significant correlation with FEV1 has been documented [234,241]. Furthermore, no significant impact of ICS treatment [37,234] or tiotropium [242] on the level of 8-isoP in exhaled air has been observed.

Considering that tobacco smoking is the primary cause of COPD, the assessment of OS markers in EBC in patients with COPD should account for tobacco smoking. Tobacco smoking is responsible for a sudden increase in OS [238,243]. The association between H_2_O_2_ and 8-isoP in EBC and tobacco smoking has been confirmed [241]. Additionally, elevated levels of 8-isoP persist in both former and current smokers with COPD, with this biomarker being more than two-fold higher in healthy smokers than in a healthy, non-smoking control group [238]. Therefore, it can be inferred that the oxidative burden in the lungs of smokers is comparable to that of COPD patients [241]. However, no significant differences were reported in H_2_O_2_ concentrations between active and former smokers in patients with COPD. Furthermore, this did not have a significant effect on H_2_O_2_ levels based on either daily or cumulative cigarette consumption [244], as well as smoking cessation duration [232]. The difference in these results may be attributed to the fact that OS resulting from tobacco smoke exposure is prominent in smokers without COPD during the initial phase, whereas inflammatory processes become more prominent as the disease progresses [37].

Malondialdehyde (MDA) is a product of the polyoxidation of long-chain fatty acids [245] and has been widely evaluated in bodily fluids as a marker of OS [246,247,248]. 

The level of MDA EBC is elevated in COPD [248,249,250] and is not dependent on the severity of symptoms [251]. Some studies did not show a correlation with disease severity (based on FEV1) [241,251], although others have confirmed not only the association of MDA with FEV1 [249,250], but also with neutrophilic inflammation [249]. The impact of COPD treatment on the concentrations of this indicator in EBC remains unclear [248,252]. 

The results of some studies suggest that MDA in EBC may be a useful tool in the early diagnosis of COPD as a differential biomarker to distinguish patients with COPD from smokers [250]. In the study by Freund et al., the level of MDA in exhaled air differentiated patients with COPD from those without COPD (*p* = 0.03), with an accuracy of 54% [253].

Nitric oxides (NOx) act as links between activated OS and NS mechanisms. An increase in exhaled air leads to elevated levels of nitrites and nitrates. Studies on NOx levels in EBC have not conclusively confirmed their correlation with tobacco smoking [45,254,255,256]. Some studies have shown increased concentrations of nitrite/nitrate in EBC among patients with COPD [257], with correlations between nitrite/nitrate levels and disease severity [112,257,258] and lung hyperinflation [259]. Elevated levels of NT have been observed in COPD patients not only in induced sputum samples [260] but also in EBC, correlating with an increase in another OS biomarker (MDA) [261]. This suggests the simultaneous activation of OS and NS stress and their potential interactions.

The results regarding NS markers remain inconsistent. Sources other than oxidative markers influence NO metabolites, such as tobacco smoking and the oral microbiome [262].

#### 4.2.3. Systemic Oxidative Stress and EBC in COPD

OS induces and stimulates inflammatory reactions, leading to COPD and cardiovascular diseases [263]. In the pathomechanism of COPD, local chronic inflammation and systemic inflammation are observed, which appear to be key determinants in the development of endothelial dysfunction in the pulmonary [264] and systemic circulation [265]. 

Chronic cardiovascular disease (CVD) is a common comorbidity of COPD [266,267]. Conversely, COPD is a documented risk factor for morbidity and mortality from cardiovascular causes [268]. The strong association between these chronic diseases was ultimately formulated in a report by the American Thoracic Society (ATS)/European Respiratory Society (ERS) working group, which recognized COPD as a pulmonary component of “multimorbidity” [269].

Shared risk factors influence the frequency of coexistence of heart failure and COPD [270]. Decompensation of heart failure is associated with alveolar lining acidification and the generation of inflammation and OS, as confirmed by EBC. This suggests that heart failure contributes to inflammatory lung damage [271]. Conversely, there have been reports of a significant relationship between inflammation markers and echocardiographic indices of right and left heart chamber function in patients with COPD. In a study by Kaźmierczak et al., it was demonstrated that serum levels of 8-isoP and LTB4, as well as IL-8 in EBC, increased, and systolic, diastolic, and overall right ventricular (RV) function deteriorated [272]. Subsequent studies have confirmed that systemic inflammation occurs in patients with increased levels of C-reactive protein (CRP), 8-isoP, LTB4, and IL-8 [273]. These mediators play crucial roles in the pathogenesis of CVD [272,274,275,276,277]. Surprisingly, the results of IL-8, LTB4, 8-isoP, and CRP levels did not differ between COPD patients with and without CVD [273], which was possibly associated with the modulating effects of statins taken by patients on inflammatory processes [278]. 

#### 4.2.4. Leukotrienes

COPD patients have been found to have elevated LTB4 levels in EBC, both in the stable and exacerbation phases [134,273,279], although EBC levels do not correlate with disease severity [280]. A reduction in LTB4 EBC was observed during convalescence [281] and after antibiotic treatment [282,283] but was not significantly associated with ICS treatment [284], which may be related to the chemotactic role of LTB4 on neutrophils in areas of inflammation [281]. Despite significantly higher LTB4 EBC levels in smokers with COPD [280], they did not distinguish active, healthy smokers from former smokers with COPD [285]. 

#### 4.2.5. Interleukins

In the proteomic analysis by Fumagalli et al., it was established that patients with COPD had a significantly higher cytokine burden than the control group, with cytokines accounting for 62% of the protein content of EBC. Among the identified cytokines were IL-1α, IL-1β, IL-2, IL-12, IL-15, interferons IFNα and γ, and TNFα [206]. The results from previous studies that identified and analyzed interleukin levels in EBC have often been conflicting, including their correlation with lung function tests (FEV1), which has also remained unclear [286,287]. Selected interleukins and cytokines assessed in EBC are presented in Table 2.

COPD is a heterogeneous and complex disease from the perspective of chronic inflammation. External factors play a role (such as tobacco smoking; occupational exposure; inhaled toxic substances; and respiratory tract infections), as well as internal factors such as oxidative and antioxidative potential; the body’s immune response; the presence of factors modulating inflammatory reactions (including tobacco smoking); and the systemic inflammatory response. These factors contribute to the difficult and precise identification of inflammatory pathways in this disease. Moreover, they lead to the development of various disease phenotypes and their overlap, further complicating the process of identifying specific inflammatory pathways. Additionally, a biomarker that could serve as a predictive factor for exacerbations in COPD has not yet been determined.

#### 4.2.6. MicroRNA

MiRNAs have the potential to serve as markers of COPD. Short description of the selected miRNA and the suspected role in the pathogenesis of COPD is presented in Table 3. They are characterized by their stability in biological fluids [291,292,293] and are easy to identify in biological materials. Previous studies on miRNAs in COPD patients have mainly focused on the identification and evaluation of blood samples [294], sputum [295,296], and EBC samples [181]. In subsequent studies, miRNAs were not only reliably detected in EBC using polymerase chain reaction (PCR) analysis but it was also suggested that ncRNAs are transmitted as signals enclosed in exosomal vesicles [297]. Among the available studies involving miRNA identification in COPD patients, only one miRNA was identified in the EBC of COPD patients [181].

NcRNA fragments play a crucial regulatory role in biological processes, and alterations in their expression promote inflammatory responses, ultimately contributing to the development of diseases such as COPD [296].

CS is a prominent cause of COPD, and the interaction between CS and miRNAs disrupts regulatory mechanisms. The CS-induced upregulation of proinflammatory factors through a feedback mechanism leads to downregulation and decreased miRNA levels [298,299]. Some studies have suggested that certain miRNAs could serve as indicators of COPD development in smokers without airflow disturbances in the respiratory system [300].

MiRNAs play significant roles in COPD development: (1) they are potent regulators of gene and protein expression; a single miRNA can interact with multiple genes simultaneously [301]; and (2) they act as modulators of chronic inflammation in COPD by influencing non-inflammatory transcription factors and altering the expression of inflammatory proteins. Exposure to CS further amplifies this response, leading to an excessive and imbalanced inflammatory process. (3) It influences the regulation of inflammatory responses by globally reducing miRNA abundance and profiling miRNA expression [299,302]. 

**Table 3:** MiRNA with a potential role in the pathogenesis of COPD. Adapted with permission from Ref. [181]- J. Allergy Clin. Immunol, Elsevier 2013;—BMJ, [301]- Thorax 2015 [303].

MiRNA	COPD	Lung Cancer	Tissue/Cell-Type	Targets	Potential Role	References
miR-146a	↓		Induced sputum, Fibroblasts	COX-2	chronic inflammation	[296,304]
	⇑		Lung tissue	COX-2	chronic inflammation	[305]
let-7c	↓		Induced sputum	TNFR-II	chronic inflammation	[296]
miR-101	↑		Lung tissue	CFTR, MPK-1	CFTR-imbalance in fluid homeostasis, MPK-1-emphysema	[306]
miR-15b	⇑		Lung tissue	SMAD7	remodeling	[305]
miR-144	⇑		Lung tissue	CFTR	imbalance in fluid homeostasis	[305]
miR-199a-5p	↑		Lung tissue	ATF6, NF-κB1, RELA, GRP78, IRE1, HIF-1α	emphysema	[307]
miR-34b	↓		Induced sputum		emphysema	[296]
miR-34c	↓		Induced sputum		emphysema	[296]
miR-34c	↓		Lung tissue	SERPINE1	emphysema	[308]
miR-638	↑/↓		Lung tissue	correlated with ≥50 predicted targets	emphysema	[309]
Let-7a	↓	↓	EBC	TGFB receptor, TLR4	remodeling	[181,310]
mirR-328	↓		EBC	IL-13, IL-15, IL-1B, IL-8, TLR2	remodeling	[181,311]
mirR-21	↓	↑	EBC	IL-13Rα, IL-17, STAT3, IL-1B	chronic inflammation remodeling, emphysema	[181,312]

↑ = increased COPD compared with never-smoker; ↓ = decreased COPD compared with never-smoker; ⇑ = increased COPD compared with smoker; [X] = corresponding reference; ATF6 = activation of transcription factor 6; CFTR = cystic fibrosis transmembrane conductance regulator; COX-2 = cyclooxygenase-2; GRP78 = glucose response protein 78; HIF-1α = hypoxia inducible factor 1α; IL = interleukin, IL-13Rα = receptor, α subunit; IRE1 = inositol requiring enzyme 1α; KCO = lung carbon monoxide transfer coefficient; Lm = mean linear intercept; MAFG = v-maf musculoaponeurotic fibrosarcoma oncogene homologue G, avian; miR = microRNA; MMP-12 = matrix metallopeptidase 12; MPK-1 = mitogen-activated protein kinase phosphatase 1; NF-κB1 = nuclear factor κB1; RELA = V-Rel avian reticuloendotheliosis viral oncogene homologue A; SERPINE1 = Serpin Family E Member 1; SMAD7 = mothers against decapentaplegic homologue 7; STAT3 = signal transducer and activator of transcription 3; TLR = toll-like receptor; TNFR-II = tumor necrosis factor receptor II.

##### Let-7

The Let-7 family plays an important role in the pathogenesis of chronic respiratory diseases [313,314], including COPD [310]. In particular, Let-7, in conjunction with its target genes, *KRAS (Kirsten Rat Sarcoma Viral Oncogene Homolog)* and EDN1, contributes to the excessive production of mucus in the airways [315]. Additionally, the absence of Let-7 has been associated with the promotion of a profibrotic phenotype in lung epithelial cells, both in vitro and in vivo, as evidenced by early fibrotic changes [316].

Reduced levels of Let-7 have been demonstrated in COPD patients, not only in blood serum [310], but also in EBC [181]. Furthermore, the decrease in Let-7 in serum was associated with the mRNA overexpression of IL-6 in peripheral lung tissues and primary bronchial epithelial cells. This suggests that the interaction of Let-7a with the target genes encoding IL-6 promotes airway remodeling [310]. In in vitro assays, IL-6 signaling activation enhanced fibroblast proliferation and extracellular matrix protein production [317], leading to increased collagen deposition in the bronchial wall [310]. Additionally, IL-6 promotes activation of the strongly profibrotic TGF-β pathway [318]. This indicates that Let-7 influences myofibroblast differentiation via the regulation of IL-6.

##### MiR-21

Elevated levels of miR-21 are present in the peripheral blood of COPD patients as well as in the lung tissue in a murine model of COPD [300]. Exposure to CS is associated with increased miR-21 expression and promotes macrophage polarization towards an M2 phenotype [312]. In contrast, Pinkerton et al. demonstrated a decrease in miR-21 levels in EBC [181]. The disparity in these results can be explained by the feedback mechanism on the axis of inflammatory factor expression and miRNAs [298,299]. 

Considering the diverse target sites of miR-21, this marker may regulate many biological pathways involved in COPD progression. In an experimental study using a murine model of COPD, increased miRNA levels in lung tissue were associated with enhanced CD68 protein expression [312], consistent with the findings of another study [319]. Additionally, in vitro studies of this marker have shown that the expression of proinflammatory cytokines in the examined macrophages may vary in different interventional environments. This suggests that macrophage transformation is variable and dynamic, and may occur between macrophage phenotypes: proinflammatory M1 and pro-apoptotic M2 [312,320]. Furthermore, the results of a study by Lu et al. suggested that an miR-21 knockout may reduce the risk of emphysema [312].

##### MiR-328

Through miRNA profiling, attempts have been made to identify miRNA panels responsible for the dysregulation of inflammatory processes in patients with COPD [181,294,321]. In a study by Pinkerton et al., significant reductions in Let-7a, miR-328, and miR-21 levels were detected in EBC compared to a healthy control group, and it was also observed that the expression of specific miRNAs could serve as a specific signature of the chronic inflammation pathway in COPD patients [181]. 

Previous findings on miR-328 and its impact on target genes have indicated its broad role in cellular biological processes. Moreover, they have been extensively evaluated in neoplastic processes that affect apoptosis [322] and proliferation [323]. Previous studies have established a link between miR-328 and lung cancer [324]. In a study by Ulivi et al., significant overexpression of miR-328 in the peripheral blood was demonstrated in patients with non-small cell lung cancer (NSCLC) compared to a group of healthy volunteers (*p* < 0.05). The high diagnostic accuracy of this marker distinguishes patients with early-stage NSCLC from healthy controls (sensitivity, 70%; specificity, 83%), thus making it a candidate marker for early lung cancer development [325]. Another study identified the regulatory function of miR-328 in the migration of cancer cells, suggesting its role as a risk factor for the development of distant brain metastases in NSCLC [324]. 

MiR-328-3p is also strongly correlated with various proinflammatory factors, such as TNF-α, IL-6, and IL-1β [185], and through its interaction with TGF-β1, it may influence the course and intensity of symptoms in obstructive airway diseases [186,187]. Additionally, a significant correlation was observed between the actin cytoskeleton pathway in respiratory epithelial cells. In in vitro tests, global silencing of miR-328 significantly delayed the pace of respiratory epithelial cell repair compared to that in control cells (*p* = 0.001), indicating a significant role of this marker in regulating respiratory epithelial wound healing [311]. 

Pulmonary hypertension (PH) is a complication of COPD, and hypoxia is the primary cause of PH development. Hypoxia leads to pulmonary vessel constriction and sustained hypertrophy, not only in the medial layer, but also throughout all layers of the pulmonary artery (PA) wall [326]. Experimental animal models have shown that hypoxia significantly reduces miR-328 expression. Conversely, miRNA-328 overexpression in mice significantly inhibits the expression of both L-type α1C calcium channels and suppresses the insulin-like growth factor 1 (IGF-1) receptor. Subsequently, apoptotic processes in pulmonary artery smooth muscle cells lead to a decrease in right ventricular systolic pressure and PA wall thickness [327]. Hypothetically, by influencing IGF-1 signaling, miR-328 also modulates the development and differentiation of various lung cell types, and a deficiency in IGF-1 signaling causes alveolar overgrowth in humans [328], suggesting the influence of IGF-1 on lung tissue architecture and emphysema development [329]. 

#### 4.2.7. The Respiratory Microbiota

Chronic exposure to tobacco smoke and environmental pollutants leads to alterations in the respiratory tract, including changes in the composition of the respiratory tract microbiota composition [330,331]. Dysbiosis of the respiratory tract microbiota influences innate and acquired immune processes and interacts with OS responses, serving as a source of oxidants [332]. Oxidants, primarily RNS, which are present in the local lung environment, can be used by bacteria as substrates for growth. This can stimulate the growth of pathogenic bacteria such as *Pseudomonas aeruginosa*, *Klebsiella pneumoniae*, *Streptococcus pneumoniae*, and *Haemophilus influenzae* [333]. The dysbiosis of lung microbiota may therefore play a significant role in the development and progression of COPD [334,335,336]. Limited data are available regarding the identification of microorganisms based on nucleic acid studies of EBC [337,338]. In a study by Zakharkina et al., microbiological results from induced sputum and EBC were compared. Only 50% of EBC samples yielded positive results, confirming the genetic material of the microorganism, with the unsuccessful detection of viral RNA. The results obtained for the two materials were not well correlated [339]. Current data evaluating microorganisms in EBC are limited, with predominantly negative results [337,338,340,341]. These unclear results may be attributed to the instability of nucleic acids in both sputum and EBC, as well as the assumption of false-positive results for upper respiratory tract flora, including *H. influenzae*, *S. aureus*, and *M. catarrhalis* [339].

### 4.3. Lung Cancer

Lung cancer is a major cause of cancer and the leading cause of cancer-related mortality worldwide [342,343]. It is estimated that, in 2020, over 2.2 million individuals were diagnosed with the disease, leading to nearly 1.8 million deaths. These statistics suggest that lung cancer is a leading cause of cancer-related death. Histologically, lung cancer is classified into non-small cell lung cancer (NSCLC) and small cell lung cancer (SCLC). NSCLC accounts for over 85% of lung cancer cases and is further categorized into three main subtypes: adenocarcinoma (AC), squamous cell carcinoma (SCC), and large cell carcinoma, with the latter being the least common subtype. Prognosis is inherently linked to the stage at which the disease is diagnosed, ranging from 90% five-year survival in stage I to 10% in stage IV of the disease [344]. 

Over the past several decades, despite advancements in awareness and knowledge of disease risk factors, the prognosis and five-year survival rate for lung cancer patients remain poor, estimated at less than 20% overall [342]. One of the reasons for this trend is the prolonged asymptomatic course of the disease, with over 55% of patients being diagnosed with metastasis [345]. In recent years, low-dose computed tomography (LDCT) has been recommended as a screening test for high-risk patients [346,347]. It is important to note that a drawback of this method is the high rate of detection of nodules (of a benign nature), which require further follow-up CT scans or biopsies. This generates costs for the healthcare system and limits access to this method in developing countries, causing discomfort and the potential risk of complications for patients undergoing additional invasive procedures.

The increasing availability of molecularly targeted therapies, particularly those dedicated to advanced NSCLC patients, has also contributed to extending survival time [345,348]. Additionally, molecularly targeted therapies have been approved as adjuvant treatments following complete tumor resection in cases of non-advanced disease (stages IB–IIIA), requiring the confirmation of specific mutations for eligibility. Hence, broadening the indications for these therapies increases the need for effective diagnostic marker testing [345].

**Figure 4:**
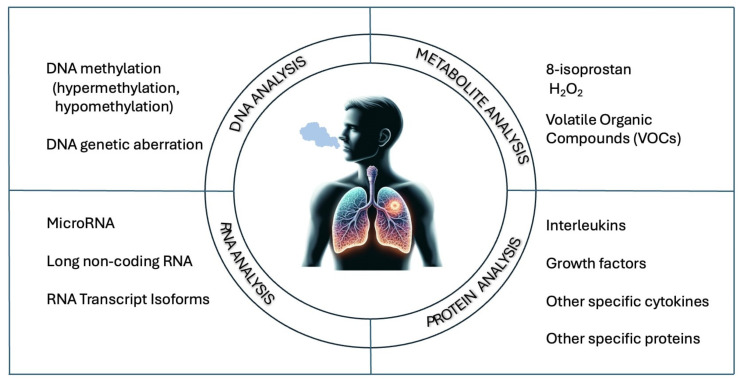
Identification of biomarkers in exhaled breath condensate for lung cancer diagnosis.

Given the global significance of lung cancer and the varied availability of diagnostic and therapeutic options, it is essential to explore new methods for improving early detection. In lung cancer detection, compounds in exhaled breath can be assessed in both gaseous form and liquid phase as exhaled breath condensate. Recently, alternative methods to EBC, such as sensor-based technologies and electronic noses (eNose) analyzing the complex patterns of gaseous exhaled breath, have been developed [349]. Lung cancer-related substances include small volatile molecules like alcohols, aldehydes, carboxylic acids, and aromatic hydrocarbons (VOCs), as well as non-volatile molecules primarily evaluated in EBC (Figure 4). Analyzing the condensate enables the detection of specific biomarkers, which is crucial for the accurate diagnosis and monitoring of lung cancer. This analysis can enhance our understanding of the disease and improve detection and monitoring methods. Below, we describe selected biomarkers that have gained significance in recent years for lung cancer diagnosis, highlighting their potential in EBC analysis.

#### 4.3.1. Epigenetic Markers of Lung Cancer in Exhaled Breath Condensate

##### MicroRNA

MiRNAs are a widely studied group of markers that arise from epigenetic changes in lung cancer. Depending on the mode of action of the molecule in the natural regulatory cycle, it may function as a suppressor or oncogene. Consequently, they affect numerous processes related to proliferation, differentiation, migration, metastasis formation, angiogenesis, and apoptosis [350,351]. They can also serve as indicators of drug resistance. Quantitative and qualitative changes in miRNA expression have been demonstrated in various types of cancer [352]. Currently, an openly accessible database called miRBase is being curated and contains information on the sequences, origins, and functions of miRNAs [353]. It encompasses nearly 2600 microRNAs that may be present in humans [354,355]. In recent years, lung cancer biomarkers have been intensively researched. MiRNAs are stable molecules present in all bodily fluids and exhaled breath. Research indicates a correlation between their content in EBC and in peripheral blood [356,357,358]. 

Among the first miRNAs identified in humans, molecules from the Let-7 family have been extensively described. Studies, including those conducted on lung cancer cell lines, have established their roles in cell cycle inhibition, consequently affecting lung cancer cell growth [359]. They inhibit the expression of oncogenes, such as *RAS* (*Rat Sarcoma Virus*, *MYC* (*Myelocytomatosis)*, and *HMGA2* (*High Mobility Group AT-Hook 2)*, which play a role in the proliferation of lung cancer cells [360]. Let-7 microRNAs directly inhibit Dicer expression (an endoribonuclease), suggesting that these molecules may also play a significant role in the production of other miRNAs [351]. In a study by Chen et al., the expression of Let-7 was compared between 30 NSCLC patients and a control group of 30 individuals [358]. Patients with NSCLC showed a significant decrease in Let-7 expression in tumor tissue, serum, and EBC compared to healthy individuals. The specificity and sensitivity of lung cancer detection in exhaled breath were 76.7% and 66.7%, respectively. No statistically significant differences were found between age groups, sex, smoking history, or histological tumor type. Interestingly, a decrease in Let-7 expression was observed in all tested samples with advanced stages of the tumor (stage I–II vs. III) and lymph node metastasis. Another study showed that the expression level of Let-7 family miRNAs (specifically reduced isoforms Let-7a and Let-7f) significantly affects the postoperative survival of patients, regardless of the initial stage of the tumor, making it an unfavorable prognostic factor [359]. In contrast to results indicating a general decrease in Let-7 expression in lung cancer, a pilot study yielded different findings [361]. In a study conducted by Rai et al., an increased expression of miR-449c, miR 31-3p, and Let-7i was detected in the EBC of lung cancer patients compared to that in healthy individuals. Previous observations have highlighted reduced levels of Let-7i in tumor tissues [362]. The authors suggested that this disparity may arise from the active release of microRNAs by tumor cells to manipulate the microenvironment and maintain their oncogenic potential.

Encouraging findings supporting the use of miRNAs in EBC for NSCLC diagnosis have been demonstrated for microRNA-186 [356]. This molecule reduces the expression of proinflammatory IL-1, which plays a role in carcinogenesis by influencing the microenvironment. Xie et al. examined 62 lung cancer patients and 60 healthy individuals, significantly proving the decreased expression of miRNA-186 in the exhaled breath and serum of lung cancer patients. A positive correlation was observed between EBC and serum. The sensitivity and specificity of the EBC analysis were determined to be 75.8% and 78.3%, respectively, with the ROC curve indicating a better marker evaluation efficiency in EBC than in serum. Interestingly, a statistically significant difference in miRNA expression in EBC between AC and SCC was noted, which may be useful for distinguishing histological types of lung cancers in the future. Additionally, a statistically significant reduction in miR-186 was observed even at an early stage of tumor progression (stage I–II), suggesting the potential use of this marker in early lung cancer detection, including in screening studies.

Numerous studies have identified potential markers of lung cancer. Mozzoni et al. analyzed the expression of miR-21 and miR-486 in EBC from NSCLC patients and a control group [357]. Significantly higher expression of miR-21 and lower expression of miR-486 were observed in patients. These differences underscore the opposing oncogenic and suppressive functions of the investigated molecules. The evaluation of the contents of both miRNAs was carried out in three types of materials: tumor tissue samples, peripheral blood, and EBC. The representativeness of these three sources was demonstrated, aligning with conclusions drawn by other researchers [356,357,358]. 

MicroRNAs can be utilized not only for lung cancer detection but also for assessing histological types. The aforementioned miR-186 showed a lower expression in patients with AC than in those with SCC. In another study focusing on adenocarcinoma, among 754 examined miRNAs, miR-597-5p and miR-1260a were identified as potential biomarkers for the early stage of this histological subtype. Unlike miR-186, both were significantly overexpressed in AC. Comparisons were also made between patients with another respiratory system tumor (mesothelioma) and those with asbestos exposure [363]. 

Current advanced technologies enable multiplex microRNA assessment in EBC, potentially enhancing early lung cancer detection efficacy. A study integrating genome-wide miRNA profiling and machine learning techniques was conducted by Pérez-Sánchez et al. showing promising outcomes [355]. Twelve miRNAs with significantly altered expression in patients with NSCLC were identified, and a profile of three markers (miR-4507, miR-6777-5p, and miR-451a) was considered optimal for patient stratification. In addition, markers distinguishing between adenocarcinoma and squamous cell carcinoma were identified. A combined panel of three miRNAs (miR-4529-3p, miR-8075, and miR-7704) improved the ability to differentiate between the two lung cancer types, achieving an AUC value of 0.98, 100% specificity, and 88% sensitivity. Disease staging and invasiveness were analyzed. Dysregulated miRNAs in EBC indicated potential target genes associated with carcinogenesis, such as *CDKN2B (Cyclin-Dependent Kinase Inhibitor 2B)*, *PTEN (Phosphatase and Tensin Homolog)*, *TP53 (Tumor Protein p53)*, *BCL2 (B-Cell Lymphoma 2)*, *KRAS*, and *Epidermal Growth Factor Receptor (EGFR)*, highlighting their potential utility in the detection, classification, and monitoring of lung cancer patients. However, as the authors acknowledge, a limitation of the study is the small sample size (42 patients, including 21 with NSCLC), underscoring the need for analysis in a larger study group.

The latest study by Shi et al. focused on the identification of 24 miRNAs selected based on lung tissue analysis and a literature review [364]. Encompassing 351 individuals, including 166 lung cancer patients, the study concentrated on miRNA analysis in EBC, taking into account detailed clinical data on chronic respiratory diseases (such as COPD and asthma) and smoking history. This study included the largest number of participants with lung cancer and EBC. Logistic regression analysis identified miRNAs with diagnostic potential, including miR-21, miR-33b, and miR-212. Employing Random Forest models that integrate clinical data and miRNAs demonstrated an enhanced ability to differentiate lung cancer by 1.1–2.5%, with the greatest benefit seen in former smokers.

In the previously mentioned pilot study by Rai et al., a broad panel of 905 miRNAs in EBC was also utilized for analysis [361]. A total of 78 significantly increased molecules were identified in this patient group, of which three (Let-7i, miR-449c, miR-31-3p) appeared to be crucial. Interestingly, these results did not align with the previously mentioned miRNA analyses. Researchers have emphasized the potential influence of external factors, such as environmental elements, chemicals, infections including tuberculosis, and the geographical region in which the study was conducted on the expression of potential biomarkers. 

It is pertinent to mention that miRNAs have been investigated not only for lung cancer diagnosis but also for therapeutic purposes. Several clinical studies have utilized synthetic versions of microRNAs to restore the natural functionality of the molecule and rectify dysregulated pathways. The first drug, MRX34, containing a version of miR-34, was used in patients with lung or primary liver cancer [365]. However, owing to the observed adverse effects in the form of immune reactions, the study was halted for safety reasons [366]. The concept of therapy using synthetic microRNAs is still evolving (e.g., Let-7, miR-16, miR-193a-3p, and miR-10b). Novel delivery methods for these molecules have been explored to minimize their side effects [367,368].

The non-invasive nature of sample collection via EBC and the stable characteristics of miRNAs suggest their potential utility as biomarkers for lung cancer. Numerous studies have demonstrated that miRNAs can serve as markers for diagnosis, histological differentiation, staging, and prognosis in patients with NSCLC. A summary of selected studies is presented in Table 4. According to recent reports, the future of miRNA research appears to be moving towards panel assessments rather than evaluations of individual molecules in EBC.

##### Long Non-Coding RNAs (lncRNAs) in Lung Cancer Research

Long non-coding RNAs are another category of molecules responsible for epigenetic changes. These molecules are more than 200 nucleotides in length and do not have protein-coding functions. Similar to miRNAs, lncRNAs can act as either oncogenic or tumor-suppressive agents, influencing the stability and translation of specific mRNAs, thereby affecting signaling pathways. Their ability to interfere with miRNAs, acting as “miRNA sponges”, potentially plays a superior regulatory role in certain instances. Although they are among the least understood RNA types, their role and diagnostic potential in respiratory diseases are being increasingly elucidated [370]. Currently, there is a limited scope of research assessing lncRNAs in EBC in lung cancer. Vardarli et al. demonstrated elevated expression of specific lncRNAs, including HOTAIR, PVT1, NEAT1, and MALAT1, in patients with lung cancer compared to healthy individuals [371]. Researchers have concluded that these lncRNAs could serve as diagnostic tools for LC detection. Furthermore, based on a comparative analysis of MALAT1 in EBC and blood, they suggested that exhaled breath might represent the molecular profile more accurately. The study population consisted solely of patients with advanced NSCLC (stages IIIB and IV). Initial analyses were performed on 40 participants, with a smaller number of patients participating in subsequent stages. 

##### RNA Transcript Isoforms as Diagnostic Biomarkers for Lung Cancer

In lung cancer diagnostics, an interesting concept of a mathematical diagnostic test based on the analysis of RNA transcript isoforms of the *GATA6* and *NKX2*-1 genes, referred to as the “LC score” has been proposed [372,373]. These genes encode proteins that are transcription factors involved in the physiological processes of lung development, repair, and cell differentiation. It has been demonstrated that in lung cancer, there are changes in the expression of embryonic and adult isoforms. In a study conducted in 2023, samples of EBC from 103 patients before the start of treatment and 23 healthy individuals were analyzed. The sensitivity of the “LC score” in detecting lung cancer in the entire study group was assessed at 92.2%, and the specificity at 82.6%. In a detailed analysis considering the stages of cancer according to the TNM classification (Tumor, Nodes, Metastasis) I, II, and III, the sensitivity of the method was determined to be 95.7%, 91.3%, and 84.6%, respectively. These results indicate a promising direction for using the EBC scheme and markers to complement current screening diagnostics for NSCLC in the future.

##### DNA Methylation

In addition to studies focused on various RNA forms, analyses of epigenetic changes in the DNA realm are being conducted. An example is DNA methylation, which is the covalent attachment of a methyl group to cytosine in DNA sequences. This mechanism governs physiological gene activity in cellular processes, but may also play a crucial role in the development and progression of diseases, including cancers. The hypermethylation of tumor suppressor gene promoters leads to inhibition of their expression, facilitating uncontrolled cell division and tumor growth [374]. On the other hand, global DNA hypomethylation can result in the excessive expression of certain genes and induce proto-oncogene activation.

EBC is a reliable material for evaluating DNA hypermethylation. Han et al. showed higher methylation densities in specific promoter regions of the *DAPK (Death-Associated Protein Kinase)* and *PAX5β (Death-Associated Protein Kinase)* genes in NSCLC patients than in healthy individuals. Additional analysis of *RASSF1A (Ras Association Domain Family Member 1)* revealed significant differences between active smokers, non-smokers, and former smokers [375]. 

Aberrant methylation patterns have also been confirmed in the p16 promoter region in patients with lung cancer. P16 is a tumor suppressor gene that plays a critical role in cell cycle regulation. Lack of or improper p16 protein expression leads to uncontrolled cell proliferation and tumor growth. In a study by Xiao et al., conducted on cancer tissue, plasma, and EBC samples from patients, disruptions were observed in 87, 50, and 40% of the materials, respectively [7]. The detection frequency of DNA hypermethylation in EBC was the lowest. As the authors indicate, to improve detection effectiveness, more rigorous sampling or procedure repetition standards may be justified. No p16 hypermethylation was detected in any individual from the healthy group, indicating the specificity of this marker for cancer. The presence of this epigenetic aberration in patients with lung cancer correlates with poorer prognosis and shorter survival time [376]. 

#### 4.3.2. Genetic Aberrations as Markers of Lung Cancer in Exhaled Breath Condensate

Genetic aberrations in lung cancer play a critical role in therapeutic selection. In recent years, the number of targeted therapies for specific molecular abnormalities has increased. Therefore, the identification of molecular disruptions such as mutations, rearrangements, and amplifications is essential. Owing to the nature of the disease, the pace of development, and the emergence of new molecular abnormalities dictating further therapy, repeated tests are necessary [345]. The Clinical Practice Guidelines of the European Society for Medical Oncology (ESMO) for advanced non-small cell lung cancer (NSCLC) driven by oncogenes in 2023 recommend molecular testing for nine predictive biomarkers [377]. The evaluation included *EGFR*, *ALK* (*Anaplastic Lymphoma Kinase)*, *ROS1 (ROS Proto-Oncogene 1)*, *BRAF (B-Raf Proto-Oncogene, Serine/Threonine Kinase)*, *MET (Mesenchymal-Epithelial Transition Factor)*, *RET (Rearranged During Transfection)*, *NTRK (Neurotrophic Receptor Tyrosine Kinase)*, *HER2 (Human Epidermal Growth Factor Receptor 2)*, and *KRAS*. Analysis is mandatory for patients with advanced non-squamous NSCLC and specific cases of squamous cell carcinoma. Tissue samples and, if necessary, liquid biopsies are the primary sources of material for testing, according to the guidelines [378]. 

However, numerous research groups have assessed the utility of EBC in detecting genetic aberrations in cell-free DNA. High concordance has been shown in the assessment of tumor tissue analysis, plasma, and EBC from NSCLC patients, particularly for *KRAS*, *EGFR, BRAF* [379,380,381,382], *PIK3CA (Phosphatidylinositol-4,5-Bisphosphate 3-Kinase Catalytic Subunit Alpha)*, and *ERBB2 (Erb-B2 Receptor Tyrosine Kinase 2)* [379]. Kordiak et al. focused on the evaluation of KRAS mutations and observed that, within the study group, EBC proved to be a more valuable sample material than blood [380]. The sensitivity and specificity of this test were estimated to be 100% and 86%, respectively. However, a limitation of the study was that the NSCLC group, in which three different source materials were compared, comprised only 19 subjects. 

On the other hand, Ryan et al. compared the results of tumor tissue analysis by Next-Generation Sequencing (NGS) with serum and EBC assessment using PCR in 125 NSCLC patients. Five oncogenic mutations controlling (EGFR, KRAS, PIK3CA, ERBB2, and BRAF) were evaluated. This study concluded that evaluating circulating tumor DNA in serum and EBC may be more useful than assessing tissue samples. This is substantiated by the fact that genetic examinations of tissue often rely on small tumor biopsy sections, leading to an incomplete tumor molecular profile due to tumor heterogeneity. Additionally, by analyzing the detailed genetic profile, certain disparities were found between the two analyzed materials. Therefore, in the future, the evaluation of both EBC and serum as complementary diagnostic methods to assess clinically significant genetic aberrations in lung cancer is recommended. Considering the EBC collection methodology, it is defined as a “lung-specific liquid biopsy” [379]. 

Some researchers have highlighted the diagnostic challenges associated with detecting DNA in collected EBC samples, attributed to specific patient inclusion criteria and preanalytical issues [383]. In a study by Youssef et al., 65.4% of EBC samples were successfully analyzed using NGS sequencing. In the remaining samples, the DNA concentration was significantly lower, resulting in ineffective amplification [384]. However, most researchers have concluded that EBC is a valuable source for molecular analysis, which, owing to its non-invasiveness, can be repeated over time, providing information on the patient’s current genetic status.

#### 4.3.3. Protein Markers of Lung Cancer in Exhaled Breath Condensate

In the quest for potential biomarkers of respiratory system malignancies, the focus has also been placed on proteins detected in exhaled air. One example is epidermal growth factor (EGF). Chen et al. analyzed 155 NSCLC cases and 115 healthy individuals and concluded that EBC-EGF levels were significantly higher in patients, with method sensitivity and specificity estimated at 80% and 89.6%, respectively (outperforming parameters concurrently assessed for EGF in blood) [385]. Among NSCLC cases, a higher level was demonstrated in advanced stages of the disease (III–IV) than in stages I–II and in patients who died within the 12-month follow-up, suggesting its utility as a prognostic factor. The usefulness of EGF in distinguishing AC from SCC has not been demonstrated. The study included a third group of patients with benign lung nodules, where the EGF levels were comparable to those in healthy individuals and significantly lower than those in NSCLS cases. This is intriguing because the current standard lung cancer screening LDCT study has limited capabilities in differentiating benign from malignant changes and entails the overdetection of changes that require further diagnostics. The measurement of EGF in these patients could aid in identifying individuals for expanded initial diagnostics.

Several studies have reported significantly higher levels of carcinoembryonic antigen (CEA) in patients with NSCLC than in healthy individuals. Remarkably, CEA may be utilized to differentiate the histologic types of lung cancer (AC vs. SCC) [386,387,388]. Parallel evaluation of endothelin-1 (ET-1) displayed similar parameters; however, its usefulness in distinguishing lung cancer types has not been demonstrated [386]. Nevertheless, a considerable reduction in EBC concentration was confirmed after successful surgical treatment, which is potentially useful in monitoring tumor regression or progression during follow-up [389]. Similar to EGF, both CEA and ET-1 levels were higher in patients with advanced stages of the disease.

An interesting aspect arises from studies evaluating CEA in EBC, which propose a miniaturized portable online detection system based on a Love-wave sensor. This system’s advantages include its low cost, ease of use, immediate bedside assessment, and integration potential with mobile phone applications (iOS) [387,390]. 

Inflammation is recognized as a key characteristic of malignant tumors, is particularly significant in the context of lung cancer, and contributes to chronic damage and inflammatory conditions. The tumor microenvironment, which consists of various cell types and the extracellular matrix, is vital for tumor growth and spread. Cytokines, including interleukins, which act as mediators between inflammation and cancer cells, play a crucial role in carcinogenesis and may serve as potential diagnostic biomarkers, interventional targets, and therapeutics [391]. They can affect the regulation of immune cell activation, which is a key transcription factor crucial for tumor progression. Considering their roles, they can be categorized into proinflammatory interleukins (IL-1β, IL-6, TNF-α) and anti-inflammatory interleukins (e.g., IL-4 and IL-10). These are additional molecules detected in the exhaled breath [392,393]. Inflammation is linked to chronic conditions, such as asthma, COPD, and obesity. Its association with carcinogenesis has also been previously established. In some studies, it served as an exclusion criterion; hence, a thorough examination of the research findings, including participant group descriptions, is crucial.

In a previous study by Xie et al., two markers, miR186 and IL-1β, were assessed in blood and EBC samples [356]. Using ELISA, increased proinflammatory IL-1β concentrations were demonstrated in both types of samples from patients with NSCLC compared to healthy individuals. Furthermore, the concentration correlated with the disease advancement stage, suggesting the potential of this marker in lung cancer diagnosis and staging. IL-1β is involved in tumor progression. Reports have emerged on the potential benefits of using the anti-IL-1β monoclonal antibody (canakinumab) to reduce lung cancer incidence and related mortality [394]. 

Increased concentrations of other inflammatory cytokines in the exhaled air of patients with NSCLC have also been confirmed: IL-6 [395,396], IL-2 [397], TNF-α [396,397,398], and VEGF [396,398,399]. Brussino et al. reported that elevated levels of IL-6, TNF-α, and VEGF were significantly correlated with tumor mass as assessed by computed tomography [396]. In the study by Gessner et al., a reduction in angiogenic markers (VEGF, bFGF—basic fibroblast growth factor) was detected in NSCLC following effective treatment [400]. Interestingly, for VEGF, bFGF, TNF-α, their potential not only to distinguish lung cancer patients (with high marker level) from healthy individuals, but also from those with benign, chronic lung diseases such as COPD, was identified [399]. This is an intriguing observation, as both diseases are associated with tobacco smoking and can coexist. In contrast to patients with chronic lung diseases (lung cancer, COPD) and healthy individuals, a significant decrease in EBC-VEGF levels occurs in acute lung injury [401]. 

Wu et al. also confirmed a significantly elevated IL-11 concentration in the exhaled breath of patients with NSCLC compared to healthy individuals, showing associations with lymph node involvement, distant metastases, disease progression, and low tumor cell differentiation [402]. IL-11 sensitivity and specificity in EBC were estimated to be 78% and 79%, respectively.

## 5. Conclusions

The EBC serves as a relatively simple, non-invasive matrix obtained by cooling and condensing exhaled air for the assessment of volatile and non-volatile biomarkers. Several commercial devices are currently available for EBC collection [59,403]. 

**Figure 5:**
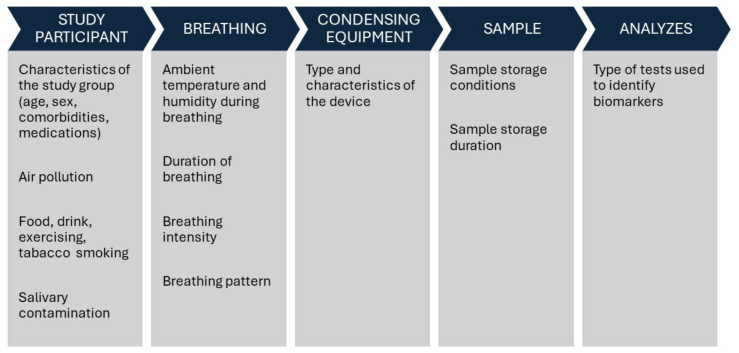
Stages of collecting EBC and factors influencing sample collection.

Breath analysis, including EBC, is emerging as a promising diagnostic tool because of its non-invasiveness, swiftness, and ability to collect samples repeatedly, which is advantageous for both patients and clinical studies. Despite the fact that EBC mainly consists of water vapor, with other chemical compounds present in trace amounts, modern highly sensitive diagnostic tests enable the effective analysis of these samples. During the collection of EBC, there are several critical moments when distortion of the objective assessment of inflammatory biomarkers is possible (Figure 5).

The American Thoracic Society (ATS) has issued guidelines regarding the measurement of EBC [19,35]. At the pre-collection stage of EBC, a common issue is the lack of detailed information on the characteristics of the studied participant group, including concurrent diseases such as obesity, smoking status, and cardiovascular conditions. These factors complicate the assessment of confounding factors and the objectivity of the EBC analysis results. Moreover, according to the Global Burden of Disease Study [404], air pollution and exposure to fine particulate matter (PM) have a significant impact on COPD, leading to worsened lung function, disease exacerbation, and an increased risk of developing and exacerbating allergic conditions [405]. Inhaled mediators may induce reactions with particles in the EBC or trigger inflammatory and/or immunological responses within the respiratory tract [406,407].

In the ERS/ATS recommendations regarding EBC collection, it is highlighted that increased exhalation flow rates during expiration reduce the efficiency of condensate collection, resulting in the dilution of the collected EBC sample [35,37]. Additionally, considerable variability in the exhaled volume occurred during EBC collection. Hence, in terms of the effectiveness of breath collection, the time of EBC collection did not determine the end of sample collection. Instead, the total volume of exhaled air dictated the endpoint and served as an indicator of the effectiveness of the EBC collection. Furthermore, it has been observed that in over 50% of studies involving EBC, there was no mention of wearing a nose clip or information regarding it [393]. Inhalation through the nose during collection can potentially contaminate the sample through several mechanisms: the mixing of biomarkers originating from the nasal cavity, nasal secretion flowing into the airways, and mingling of the nasal air fraction with the bronchial fraction. Previous reports have indicated significant differences in the assessment of exhaled biomarkers in EBC between mouth and nose breathing [40,41,408]. 

The type and characteristics of the collection device may also affect the concentration of biomarkers in the final sample [19]. In a meta-analysis of 52 studies related to 8-isoP EBC, attempts were made to establish reference norms for this biomarker, and the results additionally indicated that the stage of condensate collection is a crucial moment that affects the level of the biomarker in EBC [105]. 

According to recommendations, collected EBC samples should be promptly frozen and stored at −70 °C, and biomarker analysis should be conducted within a period when the compound under investigation remains stable [19]. The stability and reproducibility of these markers during storage are not precisely known [403]. For example, the concentration of H_2_O_2_ significantly decreases after a few days of sample storage [244,409], and prostaglandins may be destroyed by repeated freeze–thaw cycles [19]. One of the critical factors to consider is the quantification of inflammatory biomarker levels, as well as the analytical methods utilized. Commercial immunoassay kits, such as ELISA, exhibit low sensitivity and specificity for EBC [59], a matrix characterized by extremely low biomarker concentrations [36]. Results based on immunological analyses should be verified using precise analytical methods, such as LC-MS and high-performance liquid chromatography, to ensure the quantitative analysis of compounds in EBC [19,104,105]. Furthermore, there is a need to develop validated tests to identify breath biomarkers based on rapid and effective diagnostic methods.

In the context of future directions in the development of EBC biomarker research, it is worth taking into account the developing potential of CD1 molecule analysis. CD1 is an interesting molecule in the field of inflammatory processes, particularly in its role as a lipid recognizer. CD1 molecules bear a resemblance to MHC class I molecules, and the CD1 a–d isoforms are responsible for presenting both endogenous and exogenous lipid antigens. These lipids have dual functions: they act as cellular signals that initiate the development of lipid-responsive T-cell responses [410] and also play antigenic roles [411]. Ultimately, these processes affect cell proliferation, apoptosis, metabolism, and cell migration.

Endogenous lipid antigens can be identified by autoreactive lymphocytes, leading to enhanced responses when multiple lipid antigens are simultaneously presented by CD1 molecules. The expression of CD1 isoforms varies within the airways, with CD1c found in dendritic cells, monocytes, and Langerhans cells, CD1b in alveolar macrophages, and CD1d present in lung epithelial cells and classic antigen-presenting cells (APCs) [412,413].

Asthma is a chronic inflammatory disorder that is primarily characterized by the involvement of Th2 lymphocytes. However, other studies have suggested an important role for lipid-reactive classical type I invariant NK T (iNKT) cells and ILC [413] in their development. In a murine model of asthma, iNKT cells were found to play a critical role in the development of bronchial hyperresponsiveness [414]. This was demonstrated in a study in which iNKT cells were required for allergen-induced airway inflammation and hyperreactivity in an experimental asthma model. Furthermore, targeted anti-CD1c treatment abolished allergen-induced bronchial hyper-reactivity. However, it should be noted that iNKT cells did not significantly affect the development of Th2-dependent inflammation in this model [415].

In patients with asthma, the presence of dendritic cells (DCs) expressing CD1 in sputum has been associated with Th2-type inflammation [416]. Additionally, the increased expression of CD1a+ DC and CD1c+ in bronchial mucosal cells has been correlated with the number of APC cells with IL-4+ expression. This, in turn, was reflected in the abundance of IL-4 receptors in bronchial wall biopsy materials [417]. 

The significance of CD1 in COPD has been explored in various studies. In a murine model of COPD, CS exposure resulted in increased CD1 expression in DCs and alveolar macrophages. Additionally, oxidative stress within airway epithelial cells and DCs promoted iNKT activation in both the murine model and patients with COPD [418]. In this study, Pichavant et al. demonstrated an increase in CD1b expression in alveolar macrophages in smokers and patients with COPD, as assessed by EBC and/or BAL. Furthermore, there was a significant correlation between CD1b expression and disease severity measured by FEV1. Increased CD1b expression was accompanied by elevated levels of oxidative stress markers, such as 8-isoP in BAL and/or EBC and MDA in an experimental model of the disease. Moreover, as a result of lipid oxidation and consequent changes in the cellular lipid profile (lipidome) of the bronchial epithelium in COPD, the ability of macrophages to phagocytose damaged airway epithelial cells may be impaired. It should be noted that ECB was included in the methodology of this study, although not all the participants underwent ECB collection.

In the existing literature, no studies have been found on patients with asthma that incorporated an analysis of exhaled air, and data are limited in the case of COPD and EBC analyses in the context of studies on the CD1 molecule. 

Studies on the role of CD1 in lung cancer have also been conducted. It has been demonstrated that CD1d+ dendritic cells (DCs) exhibit a high expression of MHC and co-stimulatory molecules, enhancing the activation of T cells, including cytotoxic CD8+ T cells [419]. Consequently, lung cancer patients with high levels of this marker on DCs showed a stronger antitumor response, with CD1d expression positively correlated with patient survival, and its level decreasing with the advancement of cancer stages (I–IV). Similarly, in lung adenocarcinoma patients, CD1B has been identified as an independent prognostic factor [420]. High CD1B expression was associated with immune cell infiltration and immunological activation. Clinically, this correlated with a better patient prognosis and less advanced disease stages. It is suggested that CD1 may serve as a prognostic marker and therapeutic target in the future. Notably, these studies analyzed tissue samples, but not EBC. 

Numerous studies have reported the effective isolation of extracellular vesicles from various biological materials [421,422], and EBC [180,191,297]; thus, the biological material obtained during EBC collection seems to be a suitable matrix for developing this line of research. 

The non-invasiveness of EBC sampling and the stable nature of molecules such as miRNAs suggest the potential use of these biomarkers as biomarkers for chronic lung diseases, including lung cancer. However, methodological challenges, lack of standardization and validation, and a limited number of large, multicenter studies currently restrict the use of breath analysis mainly to translational research. Therefore, it is essential to conduct intensive research on the profiling and phenotyping of inflammation in asthma and COPD, as well as to detect specific biomarkers within a defined patient phenotype. Additionally, developing lung cancer risk models will enable the prioritization of screening studies in individuals at a high risk of developing the disease. 

The acceptance of EBC analysis in clinical practice requires standardization, both in terms of sample collection and interpretation. More sensitive and selective analytical techniques are needed to detect the low concentration of analytes in EBC. Standardization of EBC methods should lead to the creation of a database containing normal physiological ranges for various EBC biomarkers, which will serve as the basis for clinical diagnostics. The most sensitive methods recently developed are based on mass spectrometry, while the simplest and least efficient methods involve the use of chemical sensors or biosensor systems. Between them, in terms of cost and size, there are technologies such as laser spectroscopy. In this context, there is a real need for the development of biosensors that provide increased selectivity [423].

Cost may also be a challenge, not only related to the collection and storage of EBC samples, but also the cost of a single test, which is generated primarily by the technical requirements of highly sensitive testing methods, especially when data concerning the biomarker profile/clusters are developed.

It should be taken into account that during EBC analysis, very complex datasets are generated (often containing a huge number of compounds), which are associated with complex statistical analysis or machine learning algorithms. There is also a significant lack of population studies that would allow for the establishment of reliable discriminant factors and reference points during analysis and interpretation. 

To make EBC testing more useful in practical aspects in cases of respiratory disease, it is necessary to develop faster, real-time, more reliable, and portable tests for the presence of respiratory biomarkers [424]. Further progress in breath testing requires continuous interdisciplinary collaboration between clinicians, analytical chemists, scientists, bioinformatics experts, and industry.

In particular, the growing need for molecular and clinical predictive biomarkers to assess cancers detected in the early stages necessitates focus on integrating promising molecular and radiological biomarkers. The potential use of DNA for the systematic assessment of tumor molecular profiles in a minimally invasive manner is expanding its application in early diagnosis and prognosis as well as in disease and/or therapy monitoring in NSCLC and other cancers. 

**Figure 6:**
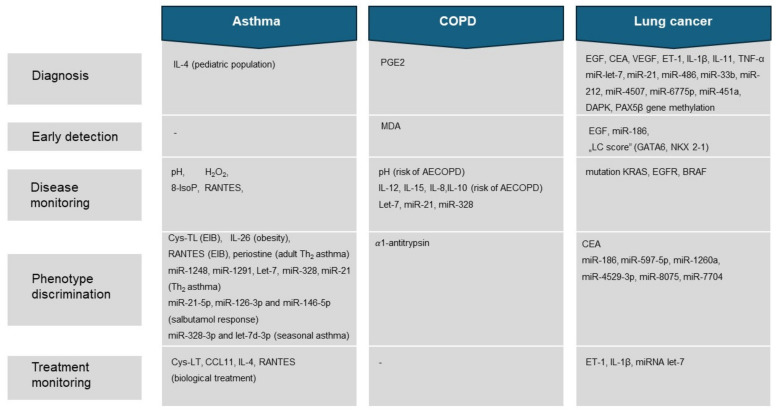
EBC markers in asthma, COPD, and lung cancer: review and proposed applications.

Figure 6 summarizes the important biomarkers analyzed thus far in EBC for asthma, COPD, and lung cancer, as well as a proposal for their potential application in clinical practice.

Despite the significant challenges in translating the latest data into clinical practice, the era of clinically utilizing reliable biomarkers is already ongoing. This objective can be achieved through rigorous standardization of sample collection, storage, processing, and analysis, ensuring the credibility and reliability of future biomarker candidates for chronic respiratory diseases.

## Figures and Tables

**Table 1:** Asthma phenotypes [49]: transitioning from clinical to molecular perspectives.

Asthma Phenotype	Main Clinical Features	Examples of Serum Biomarkers
Allergic asthma	Common onset in childhood Atopy Eosinophilic airway inflammation triggered by sputum induction Typically, a good response to inhaled corticosteroid (ICS) treatment	specific IgE, IL-4, IL-5, IL-13 [52]
Non-allergic asthma	No association with allergy Includes NERD Typically follows inflammatory pathways: Th2 and ILC2 approaches Cellular profile in induced sputum: - eosinophils - neutrophils - paucigranulocytic Poorer short-term response to ICS	IL-4, IL-5, IL-13 [52,54] Periostin [55]
Late-onset asthma (adult-onset)	Mainly women Diagnosed in adulthood Usually no allergies Often high doses of ICS	Periostin, IL-6 [56]
Asthma with persistent airflow limitation through the airways	Most likely due to remodeling Persistent airflow limitation in the airways and partially reversible air trapping [52] Mixed patterns of granulocytes with an increased number of neutrophils in the blood [56] Elevated neutrophils in the sputum [52]	Periostin, IL-6 [56] IL1B, IL-17 [57] IL-8 [52]
Asthma with obesity	Accompanying obesity Mild eosinophilic airway inflammation	Leptine, Adiponectin, IL-6, IL-10, CCL2, TNFα, Resisticine [58] Markers of oxidative stress [52]

IgE = immunoglobulin E; IL = interleukin; TNFα = tumor necrosis factor-alpha; CCL = CC chemokine ligand; [X] = corresponding reference.

**Table 2:** Selected interleukins and cytokines analyzed in EBC.

Cytokines	Stable COPD	AECOPD
IL-1B	↑ [286]	↑↑ [286]
IL-12	↑ [286]	↑↑ [286]
IL-6	↑ [288] ↓ [259]	↑↑ [286]
IL-8	⇔ [225] ↓ [259]	↑↑ [156,286] ⇔ [225]
IL-10		↑↑ [286]
TNFα	↑ [289] ⇔ [287] ↓ [259,286]	↑↑ [286]
IL-33	⇔ [290]	

↑ = increase; ↓ = reduction; ⇔ = no significant change; [X] = corresponding reference; IL = interleukin; TNFα = tumor necrosis factor-α; AECOPD = acute exacerbation COPD.

**Table 4:** MicroRNAs in exhaled breath condensate as lung cancer biomarkers: a literature review.

MicroRNA	Expression in Lung Cancer	Group (Material)	Potential Role	Reference
let-7 family:	↓	60 subjects (serum, EBC, tissue): - 30 NSCLC - 30 controls	- NSCLC detection - NSCLC staging - metastasis to lymph nodes	[358]
let-7i miR-449c miR-31-3p	↑ ↑ ↑	60 subjects (EBC): - 30 NSCLC - 30 controls	- NSCLC detection - potential marker of advanced diseases	[361]
miRNA-186	↓	122 subjects (EBC, serum): - 62 NSCLC - 60 controls	- NSCLC early detection - differentiation between AC and SCC - potential role in screening	[356]
miRNA-21 miRNA-486	↑ ↓	100 subjects (EBC, serum, tissue): - 54 NSCLC - 46 controls (coexisting other lung diseases)	- NSCLC detection	[357]
miR-4507 miR-6777-5p miR-451a	↑ ↓ ↑	42 subjects (EBC): - 21 NSCLC - 21 control group	- NSCLC detection	[355]
miR-4529-3p miR-8075 miR-7704	⇓ expression in SCC		- NSCLC differentiation between AC and SCC	
miR-6777-5p, miR-6780a-5p miR-877-5p	⇑ ⇑ ⇓		- NSCLC prognosis in 500 days’ follow-up	
miR-602 miR-551b-5p miR-1272	⇓ advanced stage (IV)		- NSCLC differentiation of stage IV from stages I–III	
miR-597-5p miR-1260a	↑	23 subjects (EBC, plasma): - 14 ACC - 9 controls	- detection of AC - differentiation with mesothelioma	[363]
miR-21 miR-33b miR212	↑	351 subjects (EBC): - 166 NSCLC - 185 controls (coexisting other lung diseases)	- enhance NSCLC detection within clinical models	[364]
miR-155	↑	30 subjects (EBC): - 15 LC - 15 controls (high risk for lung cancer)	- LC detection and staging - prognosis	[369]

↑ = elevated expression compared to control group; ↓ = reduced expression compared to control group; ⇑ = elevated expression compared to comparison group; ⇓ = reduced expression compared to comparison group; EBC = exhaled breath condensate; LC = lung cancer; NSCLS = non-small cell lung cancer; AC = adenocarcinoma; SCC = squamous cell carcinoma.
